# Inhibition of SRC-mediated integrin signaling in bone marrow niche enhances hematopoietic stem cell function

**DOI:** 10.1016/j.isci.2022.105171

**Published:** 2022-09-19

**Authors:** Irene Mariam Roy, P.V. Anu, Samantha Zaunz, Srinu Reddi, Aravind M. Giri, Rithika Saroj Sankar, Sarah Schouteden, Joerg Huelsken, Catherine M. Verfaillie, Satish Khurana

**Affiliations:** 1 School of Biology, Indian Institute of Science Education and Research Thiruvananthapuram, Thiruvananthapuram, Kerala 695551, India; 2 Stem Cell Institute, KU Leuven, 3000 Leuven, Belgium; 3 École Polytechnique Fédérale de Lausanne (EPFL), 1015 Lausanne, Switzerland

**Keywords:** Biological sciences, cell biology, stem cells research

## Abstract

Interaction with microenvironmental factors is crucial for the regulation of hematopoietic stem cell (HSC) function. Stroma derived factor (SDF)-1α supports HSCs in the quiescent state and is central to the homing of transplanted HSCs. Here, we show that integrin signaling regulates *Sdf-1α* expression transcriptionally. Systemic deletion of Periostin, an Integrin-αv ligand, showed increased expression of *Sdf-1α* in bone marrow (BM) niche. Pharmacological inhibition or CRISPR-Cas9-mediated deletion of SRC, resulted in a similar increase in the chemokine expression *in vitro*. Importantly, systemic SRC-inhibition led to increase in SDF-1α levels in BM plasma. This resulted in a robust increase (14.05 ± 1.22% to 29.11 ± 0.69%) in the homing efficiency of transplanted HSCs. In addition, we observed enhancement in the recovery of blood cell counts following radiation injury, indicating an enhanced hematopoietic function. These results establish a role of SRC-mediated integrin signaling in the transcriptional regulation of *Sdf-1α*. This mechanism could be harnessed further to improve the hematopoietic function.

## Highlights

• Outside-in integrin signaling regulates *Sdf-1α* expression
• SRC-inhibition increases *Sdf-1α* expression in osteo- and mesenchymal lineage
• Saracatinib-induced elevated SDF-1α level in BM plasma increases HSC homing
• Higher SDF-1α levels aid radiation resistance and hematopoietic recovery

## Graphical abstract

**Figure:**
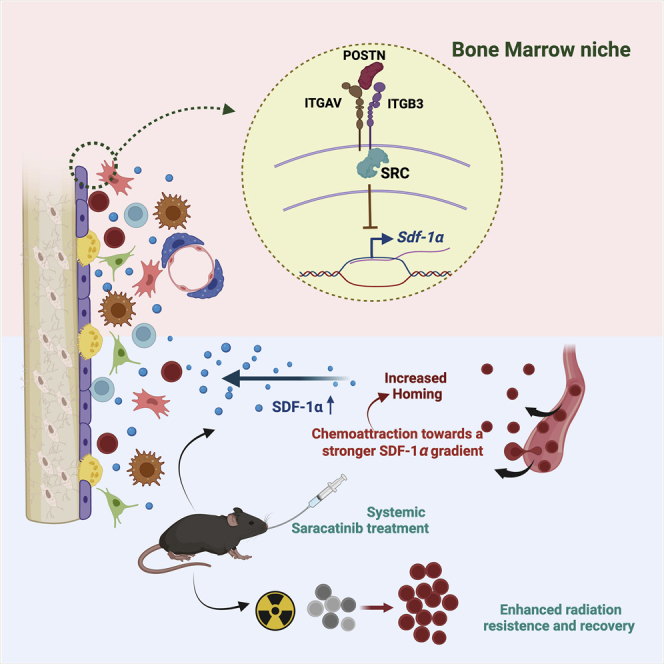

## Introduction

Hematopoietic stem cells (HSCs) are responsible for the continuous production of all required blood cell types in a specific proportion, determined by the physiological status of an organism (Adams and Scadden, 2006). It has been well-established that in conjunction with HSC-intrinsic factors, the microenvironment regulates cell fate decisions in HSCs (Boulais and Frenette, 2015). Cell membrane receptors mediate the effects of microenvironmental factors and provide physical and molecular support for HSC function (Wilson and Trumpp, 2006). Integrins belong to one such important class of heterodimeric proteins that mediate cellular attachment to ECM components (Streuli, 2009). Through conformational changes regulated by inside-out signaling mechanisms, integrins mediate physical maintenance of adult HSCs in the bone marrow (BM) niche (Suda et al., 2005). Various integrins, such as Integrin-α4β1 (Leavesley et al., 1994) (Ramirez et al., 2009), Integrin-α5β1 (Kerst et al., 1993), and VLA6 (Notta et al., 2011), were found to be crucial for hematopoietic function. For example, the neutralization of VLA4 using antibodies led to the mobilization of hematopoietic progenitors (Papayannopoulou and Nakamoto, 1993). Our results showed that enhancement in VLA4 expression can overcome culture-induced loss of homing potential (Khurana et al., 2013a). Cumulatively, inside-out integrin signaling-mediated HSC interaction with their niche has emerged as the key for physical retention and homing upon transplantation (Quesenberry and Becker, 1998). Contrarily, the role of outside-in integrin signaling in hematopoiesis has not been well elucidated.

Integrin heterodimer -αvβ3 (ITGAV-B3), a receptor for a variety of ligands such as Periostin (POSTN), Osteopontin (OPN) and so forth, has been shown to elicit outside-in signaling in various cell types (Suresh et al., 2019). It was reported to be differentially expressed in the HSC population (Umemoto et al., 2006) (Umemoto et al., 2008), which we later showed was important for the regulation of proliferative activity in adult HSCs (Khurana et al., 2016). We extended these studies and recently reported that Postn-Itgav interaction plays a role in the function of splenic HSC function (Mehatre et al., 2021). Furthermore, this study showed the involvement of POSTN in splenic HSC niche formation as *Postn*-deletion led to reduced support for the incoming HSCs in spleen, upon transplantation. Importantly, the effect of POSTN-ITGAV-mediated signaling in the regulation of HSC proliferation was found to be consistent, irrespective of developmental stage (Biswas et al., 2020). Although studies have established the role of ITGB3-containing integrin heterodimers in outside-in signaling, it has also been shown to mediate adhesion dependent inside-out signaling events elicited by thrombopoietin (Umemoto et al., 2012). This study also showed that ITGB3 is involved in the maintenance of long-term repopulation activity of HSCs. Contrary to this, another report suggested a suppressive effect of ITGAV-B3 on HSC function that assists the effects of IFN-γ on the hematopoietic system (Umemoto et al., 2017).

The expression of ITGAV as well as ITGB3 has been reported in a variety of cell types that are closely associated with HSC niche. ITGAV (CD51) is used as a common marker to identify mesenchymal stem cells (MSCs) as well as osteoblasts (Pinho et al., 2013). ITGAV-mediated integrin signaling regulates a variety of functions, such as differentiation and migration, in MSCs (Matsuzawa et al., 2015) (Olivares-Navarrete et al., 2015). ITGAV-B3 heterodimer also mediates BMP-2 induced osteoblast differentiation via ILK/ERK pathway (Su et al., 2010). In addition to regulating cell fate decisions, integrin signaling through ITGAV was shown to mediate the response to mechanical stimulation in osteoblasts (Weyts et al., 2002). Importantly, its ligand POSTN has been involved in diverse physiological processes in osteoblasts, and impacts bone remodeling (Cobo et al., 2016). Owing to the diverse functions of this ligand-receptor pair, a broad expression pattern has been reported. The resulting signaling events impact cell migration and metastasis in several types of cancers (Gillan et al., 2002) and cardiac healing following ischemia (Shimazaki et al., 2008). Through an shRNA screen, an involvement of both ITGAV and ITGB3 was demonstrated in leukemogenesis using MLL-AF9 acute myeloid leukemia (AML) model (Miller et al., 2013). Despite a clear role of integrin signaling in regulating a number of cellular functions, there has not been a consistent demonstration of the pathways involved and gene-sets directly under the control of integrin signaling.

Several secretory niche factors play important roles in the functional regulation of HSCs (Nakahara et al., 2019). Some of them, such as SCF and SDF-1α, have been used to identify the niche components crucial for the HSC maintenance and function (Ding et al., 2012) (Ding and Morrison, 2013). SDF-1α has emerged as a key factor that determines the physical maintenance of HSCs in their niche through interaction with its receptor CXCR4 (Sugiyama et al., 2006). It is well established that the SDF-1α gradient is essential for homing and engraftment of transplanted HSCs (Yin and Li, 2006). Others have shown that the disruption of SDF-1α-CXCR4 interaction in BM niche leads to the migration of HSCs from BM to peripheral blood (Zhang et al., 2014). We earlier showed that increasing the expression of *Sdf-1α* increases homing efficiency and engraftability of transplanted hematopoietic stem and progenitor cells (HSPCs) (Khurana et al., 2014). Consistent with this, temporal over-expression of *Sdf-1α* led to increased hematopoietic function, reflected in better radiation rescue (Rajendiran et al., 2020). Therefore, efforts to understand the regulation of *Sdf-1α* expression were made that resulted in the identification of transcription factors that could transactivate *Sdf-1α* promoter (Calonge et al., 2010). However, the *in vivo* relevance of these observations in the regulation of hematopoietic function has not been clear.

Here, we implicate integrin signaling in the transcriptional regulation of *Sdf-1α* expression in HSC niche. Mice lacking *Postn* expression showed higher level of *Sdf-1α* transcripts in the non-hematopoietic fraction of BM cell population. The expression of *Sdf-1α* could be increased via inhibiting Src phosphorylation, *in vitro* as well as *in vivo*. Elevated *Sdf-1α* expression resulted in enhanced homing of incoming transplanted HSCs. It also led to faster recovery from radiation injury, albeit causing no significant change in long-term engraftment potential of the stem cell population. These findings present evidence for the involvement of c-Src-mediated integrin signaling in the transcriptional regulation of *Sdf-1α*, relevant to the ongoing efforts to improve HSC engraftment.

## Results

### Inhibition of Src phosphorylation increases *Sdf-1α* in BM stromal cells

**Figure 1:**
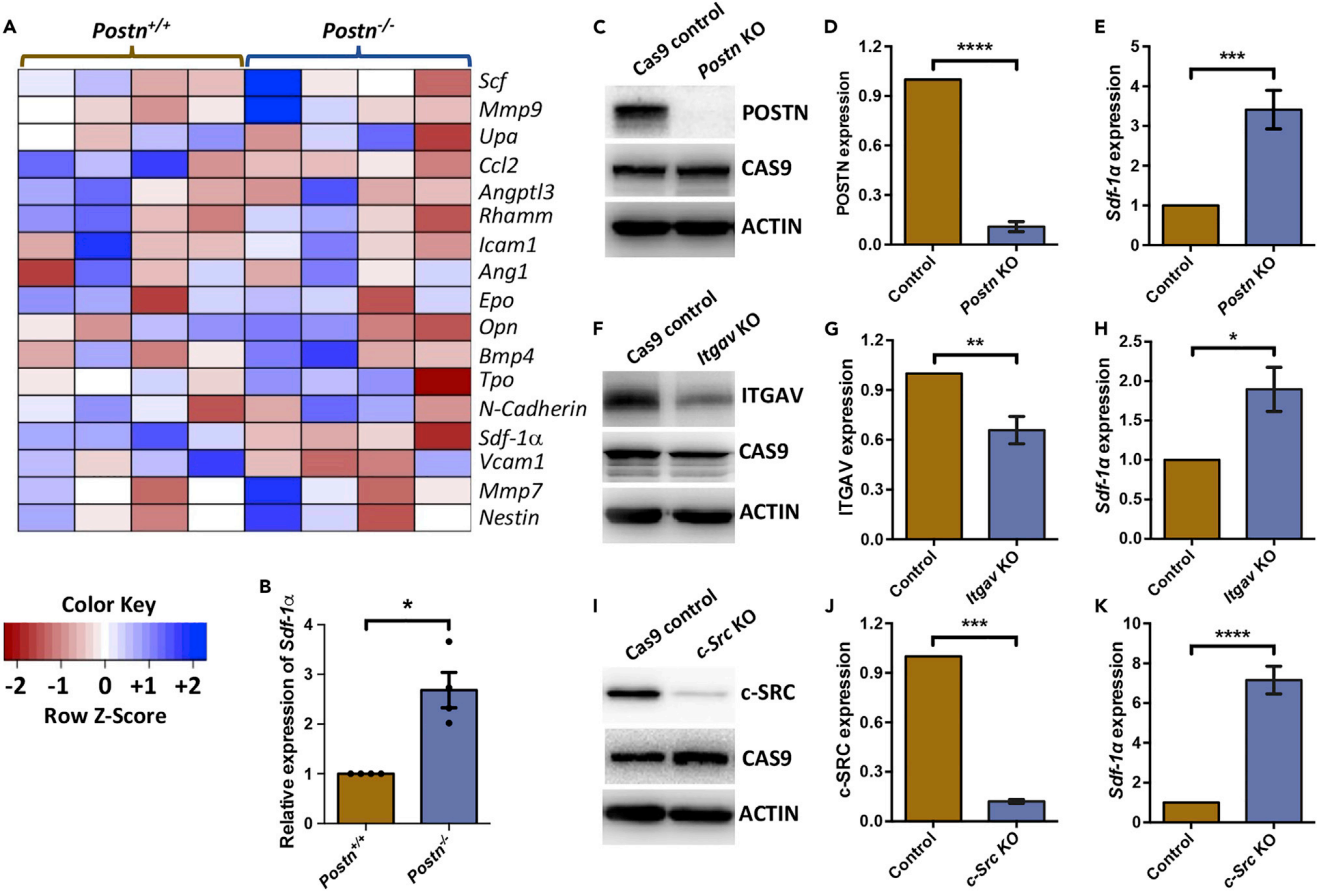
Inhibition of Integrin and c-Src phosphorylation increases *Sdf-1α* in the BM stromal cells (A) Quantitative RT-PCR experiments were performed to examine the expression of known regulators of HSC function. Heatmap represents relative expression levels of various genes in BM derived lin^−^CD45^−^ cells from *Postn*^*+/+*^ and *Postn*^*−/−*^ mice. (B) Relative expression (fold change) of *Sdf-1α* in lin^−^CD45^−^ BM cells from *Postn*^*+/+*^ and *Postn*^*−/−*^ mice by qRT-PCR. n*=*4; Mann-Whitney test: ∗p < 0.05. (C) Immuno-blotting for POSTN, CAS9 and actin expression in *Postn*-KO ST2 cells and the control cells expressing CAS9. (D) Comparison of POSTN expression by densitometry analysis of the immunoblots. Immunoblotting was performed for POSTN in CAS9 control and *Postn* KO cells. Data normalized with actin expression is presented as average values with the error bars representing SE mean. *n=*9; t-test: ∗∗∗∗p < 0.0001. (E) Relative expression of *Sdf-1α* expression in *Postn* KO ST2 cells in comparison to CAS9 control, examined by performing quantitative RT-PCR experiments. *n=*10; t-test: ∗∗∗p < 0.001. (F) Representative blots from immunoblotting experiments performed to compare the expression of ITGAV, CAS9 and actin in CAS9 control and *Itgav* KO ST2 cells. (G) Comparison of ITGAV expression by densitometric analysis of immunoblots from experiments performed on CAS9 control and *Itgav* KO cells. Arbitrary values obtained from ITGAV blots were normalized with actin. Data is represented as average ±SEM after the analysis of six biological replicates using Student’s *t* test. *n=*6; t-test: ∗∗p < 0.01. (H) Relative expression of *Sdf-1α* in *Itgav* KO ST2 cells in comparison to CAS9 control. The expression was analyzed by performing quantitative RT-PCR. n*=*7; t-test: ∗p < 0.05. (I) Immunoblotting experiments to check the deletion of SRC protein expression in *c-Src* KO ST2 cells and to confirm CAS9 expression. Actin expression was used as internal control. (J) Quantification of SRC protein expression from immunoblots by densitometry analysis, normalized with actin expression. Data is presented as average values with error bars representing SEM from three independent biological replicates. *n=*3; ∗∗∗p < 0.001 by Student’s *t* test. (K) Relative fold change of *Sdf-1α* expression in c-Src KO ST2 cells in comparison to CAS9 control. *n=*8; ∗∗∗∗p < 0.0001, Mann-Whitney test.

We have reported earlier that the lack of POSTN-ITGAV interaction leads to faster cycling and exhaustion of adult BM HSCs. In addition to the hematopoietic cell-specific deletion of *Itgav*, systemic deletion of *Postn* had shown an increase in the proliferative activity of HSCs. To examine if systemic deletion of *Postn* had any niche-mediated effects on the hematopoietic system, we tested the expression of known hematopoietic regulators in the non-hematopoietic fraction of BM cells. We isolated lin^−^CD45^−^ fraction of BM cells from *Postn*^*−/−*^ mice and performed quantitative RT-PCR to examine the expression of genes known to be expressed in the BM niche cells (Figure 1A). Results clearly showed an increase in the transcript level of *Sdf-1α* (Figure 1B), *Vcam1* (Figure S1A) and *Ccl2* (Figure S1B) in *Postn*-deficient mice. The most prominent of these, *Sdf-1α*, showed a 2.68 ± 0.36-fold increase (Figure 1B; n = 4, p = 0.003) in the cells isolated from *Postn*^*−/−*^ mice than the WT controls. As POSTN has been shown to bind most prominently to -αvβ3 integrin heterodimer, we examined the expression of the two sub-units (αv as CD51, and β3 as CD61) in various BM niche cells by immuno-staining followed by flow cytometry analysis (Figures S1C-S1E). The non-hematopoietic cell population (lin^−^CD45^−^ MNCs from BM) was further gated for CD31^+^ cells to identify endothelial cells (Figure S1C), and LepR^+^ cells as perivascular stromal cells (Figure S1D). In addition to these, PDGFR-α and Sca-1 staining was performed to identify PαS cells, MSCs and osteoblast progenitor (OP) cells (Figure S1E). Consistent with earlier reports, we found expression of both integrin sub-units, albeit modest.

In order to further confirm these results, we targeted the intrinsic expression of *Postn* in the adult mouse BM-derived stromal cell line ST2 using CRISPR-Cas9 system. As expected, we noted almost complete lack of POSTN expression in these cells (Figures 1C and 1D) after the introduction of three different gRNAs against exon 1 and 4 of *Postn* gene (Table S1). Furthermore, the analysis of *Sdf-1α* expression by qRT-PCR revealed a clear increase (Figure 1E). POSTN exerts its effects on a number of physiological functions through interaction with ITGAV (Mishra et al., 2020). Therefore, we tested if ITGAV-mediated transcriptional regulation of *Sdf-1α* expression. Three gRNAs were designed against exon 5 and 6 of *Itgav* gene (Table S1), and we confirmed a significant decrease in the expression of ITGAV (Figures 1F and 1G). This partial deletion of the gene, most cellular heterogeneity, was enough to demonstrate a clear increase in the expression of *Sdf-1α* (Figure 1H).

SRC family protein tyrosine kinases (SFKs) are central to the downstream events in ligand-mediated outside-in integrin signaling. ITGB3 that partners with ITGAV in a heterodimeric association to generate the receptor for POSTN, has been shown to directly interact with SRC (Shattil, 2005). Following these lines, we examined the role SRC in mediating the effects of outside-in integrin signaling in the regulation of *Sdf-1α* expression. Exon 7 and 10 of *Src* gene were targeted using three gRNAs (Table S1) and gene deletion was obtained resulting in almost complete abrogation of protein expression (Figures 1I and 1J). Importantly, we observed an increase in the expression of *Sdf-1α* (Figure 1K), which was higher than the change that resulted following the deletion of *Postn* (Figure 1E) or *Itgav* (Figure 1H). These results confirm the involvement of c-Src-mediated outside-in integrin signaling in the regulation of *Sdf-1α* expression.

### Inhibition of integrin signaling improves chemo-attraction properties of stromal cells

**Figure 2:**
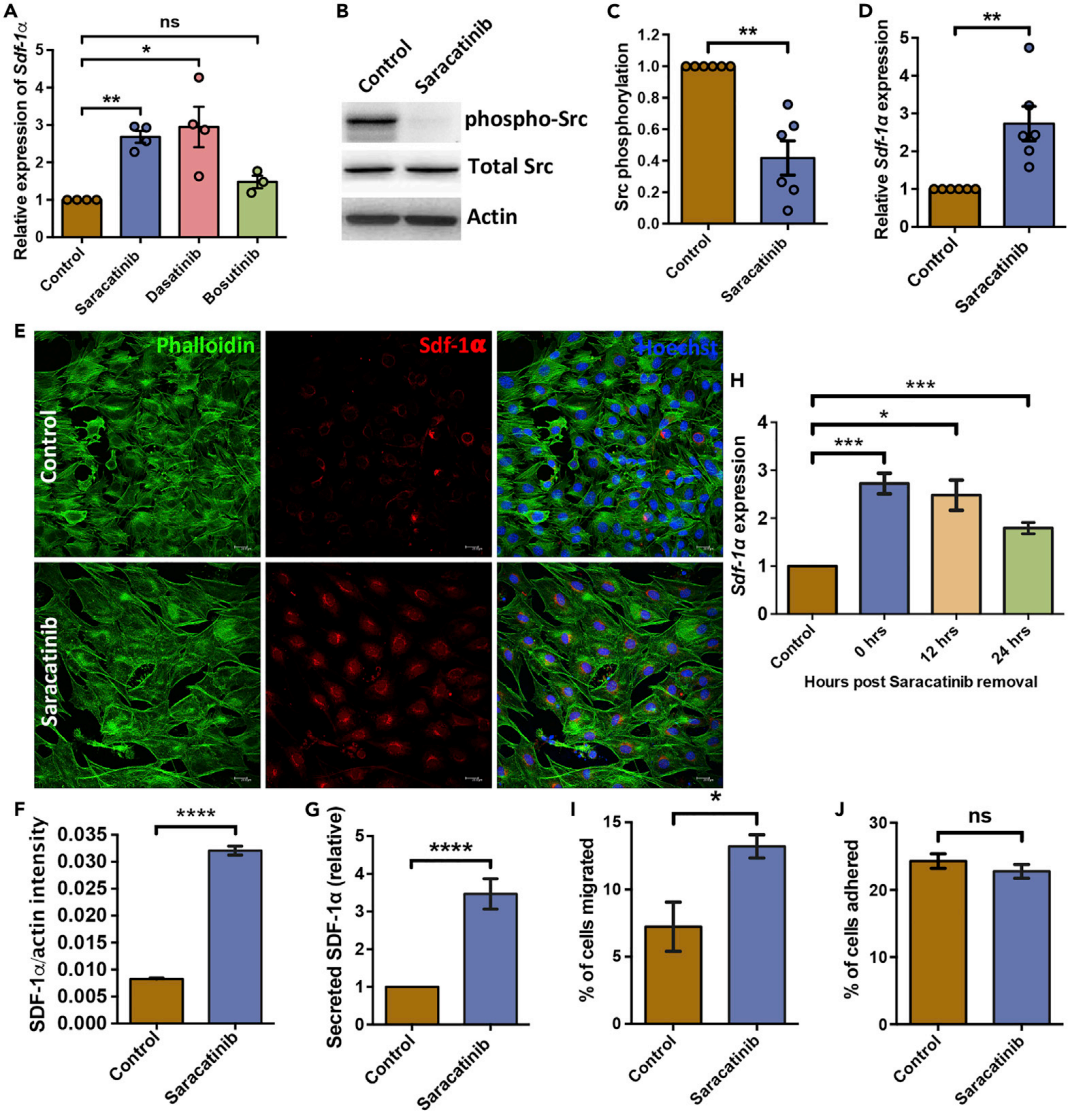
Increase in Sdf-1α expression is correlated with increased chemo-attraction of HSPCs (A) Relative expression of *Sdf-1α* in ST2 cells treated with 2μM Saracatinib (n*=*4), 150nM Dasatinib (n = 5), or 5μM Bosutunib (n = 5) in comparison to carrier control (fold change presented). t test ∗p < 0.05, ∗∗p < 0.01, ns represents not significant p > 0.05. (B) Comparison of the phosphorylation status of SRC protein in ST2 cells cultured with or without 2μM Saracatinib for 48 h. Total SRC protein and actin were used as internal controls. (C) Densitometry analysis of immunoblots to compare the phosphorylation status of Src. Normalization was conducted against actin levels by Quantity One software. *n=*6, Mann-Whitney test ∗∗p < 0.01. (D) Relative *Sdf-1α* expression in primary bone marrow stromal cells cultured for 48h with or without Saracatinib. *n=*6; t test: ∗∗p < 0.01. (E) Detection of intracellular SDF-1α levels in ST2 cells after 48h of culture with (lower panel) or without (upper panel) Saracatinib. Immuno-staining was performed using monoclonal antibodies against SDF-1α. Hoechst 33,342 and Phalloidin dye were used to label nuclei and actin cytoskeleton, respectively. Scale bar - 20.6 μm (F) Comparison of intracellular SDF-1α levels in ST2 cells cultured with or without Saracatinib. Fluorescence intensity for SDF-1α signals was normalised with phalloidin (Actin). *n=*3, *N=*2400-2600 cells, t test: ∗∗∗∗p < 0.0001. (G) Level of SDF-1α in culture supernatant as a measure of protein secreted by the ST2 cells. The cells were cultured for 48h with or without Saracatinib and ELISA experiments were performed on culture supernatant. *n* = 6; t test: ∗∗∗∗p < 0.0001. (H) Relative expression of *Sdf-1α* in ST2 cells, post-removal of 2μM Saracatinib. Quantitative RT-PCR experiments were performed after different time points after the Saracatinib treatment of ST2 cells. n = 6-7, t test: ∗p < 0.05, ∗∗∗p < 0.001. (I) Comparison of chemo-attractive property of ST2 cells treated with Saracatinib. *In vitro* migration assays performed using HSPCs in *trans*-well chambers, separated from Saracatinib treated or untreated ST2 cells by 3μm pore size membrane. Relative proportion of PKH-26 labeled HSPCs that migrated toward ST2 cells within 3h was quantified by flow cytometric analysis. *n=*4; t test: ∗p < 0.05. (J) *In vitro* adhesion assays performed to quantify the attachment of PKH-26 labeled HSPCs on ST2 cells within 3h. ST2 cells treated with vehicle or Saracatinib were compared for the attachment of HPSCs by performing flow cytometry. Relative proportion of HSPCs adhered to ST2 cells was plotted. *n=*6; t test: ns not significant p > 0.05.

In order to further understand the involvement of integrin signaling in niche-mediated regulation of hematopoietic function, we decided to use pharmacologic inhibition of Src-mediated signaling. We used three different Src inhibitors; Saracatinib (2μM), Dasatinib (150nM) and Bosutinib (5μM) and tested their effect on *Sdf-1α* expression (Figure 2A). Both Saracatinib and Dasatinib increased the transcript levels of *Sdf-1α* in ST2 cells, while Bosutinib was not equally effective. Owing to lesser off-target effects and a consistent impact on the expression of *Sdf-1*α, we chose Saracatinib for all further experiments. We tested the effect of different doses of Saracatinib on *Sdf-1α* expression in ST2 cells (Figure S2A) and chose 2μM concentration for our experiments. Furthermore, based on examination at different timepoints, we noted that a 48h culture period showed the highest impact on *Sdf-1α* expression (Figure S2B). The inhibitory effect of Saracatinib on Src activation was confirmed by immunoblotting (Figures 2B and 2C). Using phospho-specific antibodies (Tyr416), we detected a clear decrease in the phosphorylation of Src kinase. Quantification was performed using densitometry, where phospho-Src levels were normalized with actin levels (Figure 2C). We then examined if the effect of Src inhibition had the same effect on *Sdf-1α* expression in primary BM stromal cells. The stromal cells from mouse BM were treated with 2μM Saracatinib and expression of *Sdf-1α* was examined by performing quantitative RT-PCR (Figure 2D). We noted that the effect of Src inhibition on *Sdf-1α* expression remained the same, as we observed a significant increase (2.73 ± 048-fold, n = 6, p = 0.002) in its expression. To assess the functional relevance of elevated *Sdf-1α* transcript level, we first tested its protein expression and secretion. We also performed immuno-staining experiments to confirm the increase in the protein level of SDF-1α (Figure 2E). Monoclonal antibodies against Sdf-1α were used to immuno-label ST2 cells, cultured with or without Saracatinib, along with actin staining using Phalloidin. Fluorescence intensity of SDF-1α signals was normalized against actin staining at single cell level using Cell Profiler, and compared between Saracatinib treated and untreated samples (Figure 2F; 0.008 ± 0.00021 in control versus 0.032 ± 0.0008 in Saracatinib treated). Results clearly showed an increased level of Sdf-1α protein expression following the inhibition of integrin signaling. We also performed ELISA to examine its levels in the culture supernatant following Saracatinib treatment in comparison to the control (Figure 2G). We observed a significant increase in SDF-1α secretion from Saracatinib treated ST2 cells, in comparison with control (3.46 ± 0.59-fold; n = 6, p = 0.002). After confirming that the inhibition of Src phosphorylation leads to an increase in the expression and secretion of SDF-1α, we examined if it had any effect on the adhesive or chemo-attractive properties of stromal cells. These experiments were based on contact or non–contact-based culture, respectively. In order to avoid exposure of hematopoietic cells to Saracatinib, we washed the stromal cell layer. Therefore, prior to these assays, we tested if the altered expression of *Sdf-1α* was maintained even after Saracatinib removal. For checking this, we treated ST2 cells with Saracatinib and examined the expression of *Sdf-1α* 12 and 24h after washing the cells (Figure 2H). The results showed that the elevated level of *Sdf-1α* seen after Saracatinib treatment was maintained at least upto 24h after washing the cells (Figure 2H). Subsequently, we performed *in vitro* migration assays using *trans*-well chambers. In these experiments, the hematopoietic progenitors are allowed to migrate vertically toward the chemo-attractant source. The proportion of lineage depleted BM cells, labeled with membrane binding fluorescent dye PKH-26, that migrated to the ST2 cells, cultured with or without Saracatinib was examined by flow cytometry (Figure 2I). We observed a clear increase in the proportion of BM cells that migrated to the ST2 cells following the inhibition of integrin signaling (7.23 ± 1.83 to 13.21 ± 0.86, n = 4, p = 0.028). In adhesion assays, the HSPCs were labeled with PKH-26 and co-cultured with ST2 cells, treated with or without Saracatinib. After 3h, the non-adherent cells were removed and the ST2 cells along with the adhered hematopoietic progenitors were harvested following an additional wash. The proportion of total cells plated that adhered to the stromal cell layer was enumerated by flow cytometry analysis (Figure 2J). We did not observe any change in the proportion of hematopoietic progenitors that adhered to the Saracatinib treated ST2 cells, compared to untreated cells.

### Modulation of integrin signaling affects cell cycle status and expands the phenotypic hematopoietic stem cell compartment

**Figure 3:**
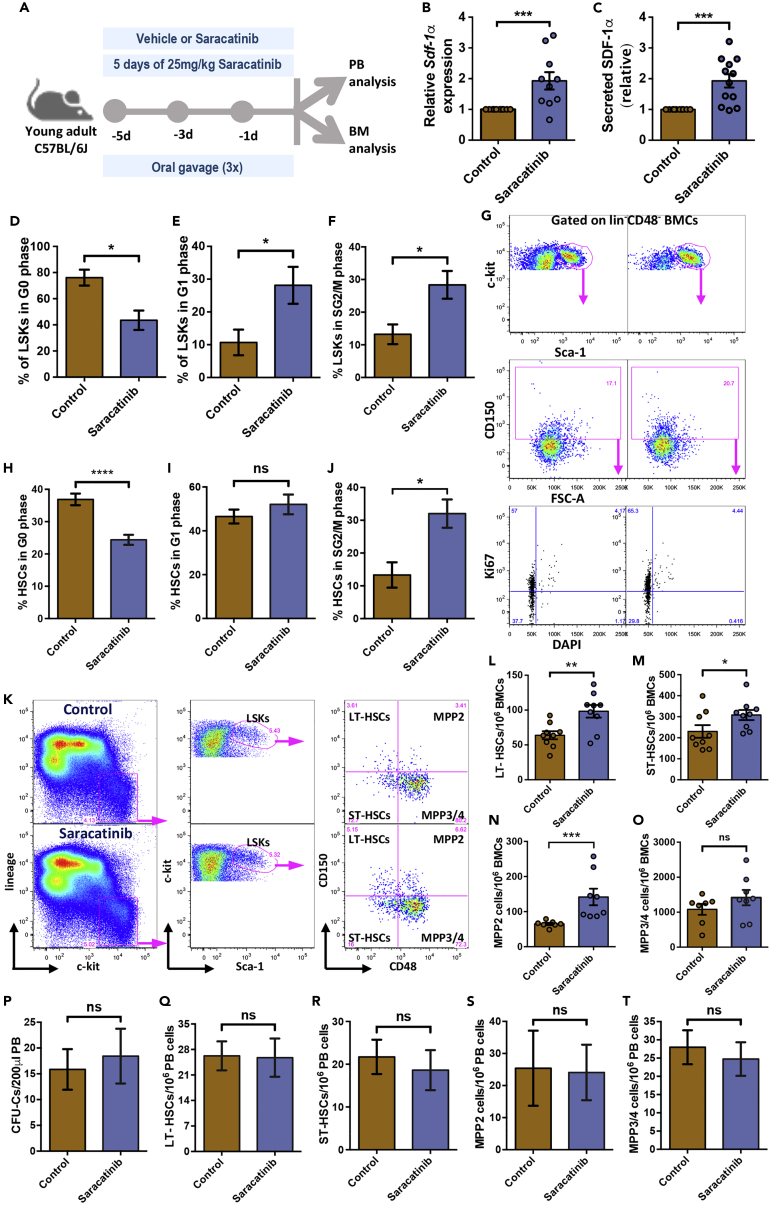
Treatment with Src inhibitor Saracatinib alters cell cycle status of HSCs *in vivo* (A) Schematic representation of the experiments performed to examine the effect of Saracatinib on HSC cycling and differentiation. Three doses of vehicle alone or Saracatinib (25 mg/kg), through oral gavage, were given on alternate days followed by hematology analysis of PB cells and flow cytometry of BM MNCs. (B) Quantitative RT-PCR analysis performed to quantify *Sdf-1α* transcript levels lin^−^CD45^−^ BM cells from vehicle or Saracatinib treated mice. *Actb* was used as the internal control and relative gene expression was plotted. *n=*5, *N=*10; Mann-Whitney test: ∗∗∗p < 0.001. (C) Comparison of SDF-1α secretion in the BM plasma of vehicle or Saracatinib treated mice. The samples from the two groups of mice were used for ELISA-based detection and quantification. Relative levels of protein in the two groups of samples is shown. *n=*6, *N=*12; t test: ∗∗∗p < 0.001. (D-F) Flow cytometry-based cell cycle analysis of the LSK cells from the BM MNCs of vehicle and Saracatinib treated mice. The LSK cells were analyzed for 7-AAD and Ki67 staining to quantify the proportion of cells in G_0_ (D), G_1_ (E), and SG_2_/M (F) stages of cell cycle. *n=*6, *N=*6-8; Mann Whitney test: ∗p < 0.05. (G) Flow cytometry plots for the analysis of cell cycle stages of HSC cells from the BM MNCs of vehicle and Saracatinib treated mice. The Sca-1^+^c-kit^+^ cells gated on lin^−^CD48^−^ cells (top panel) were further gated for CD150^+^ cells identifying the adult BM HSCs (middle panel). The HSCs were analyzed for DAPI and Ki67 staining to analyze the proportion of cells in different cell cycle stages. (H-J) Comparison of the proportion of HSCs in G_0_ (H), G_1_ (I), SG_2_/M (J) stages of cell cycle in control and Saracatinib treated mouse BM. n*=*4, *N=*8; Mann-Whitney test: ∗p < 0.05, ∗∗∗∗p < 0.0001, ns not significant. (K) Flow cytometry-based analysis of BM cells to quantify the frequency of BM HSCs in vehicle and Saracatinib treated mice. The BM lin^-^c-Kit^+^ cells gated for Sca-1^+^ cells (LSK cells) were further analyzed for CD48 and CD150 expression for detecting LT-HSCs (CD48^−^CD150^+^ cells; upper left), ST-HSCs (CD48^−^CD150^-^ cells; bottom left), MPP2 (CD48^+^CD150^+^ cells; upper right), and MPP3/4 (CD48^+^CD150^-^ cells; bottom right). (L-O) Frequency of LT-HSCs (L), ST-HSCs (M), MPP2 (N), and MPP3/4 (O) among LSK cells in the BM of the mice treated with or without Saracatinib. Flow cytometry-based experiments were performed on BM MNCs, and quantifications were performed based on gates shown in adjacent panels (K). *n=*9 Mann-Whitney test: ∗p < 0.05; ∗∗p < 0.01, ∗∗∗p < 0.001. (P) Comparison of the circulating hematopoietic progenitors in peripheral blood following Saracatinib treatment. Methylcellulose-based colony assays were performed to detect CFU-Cs in 200μL peripheral blood from Saracatinib treated or untreated mice. n = 3, t test: ns not significant p > 0.05. (Q-T) Flowcytometry-based detection of circulating hematopoietic stem and progenitor cells. Frequency of LT-HSCs (Q), ST-HSCs (R), MPP2 (S), and MPP3/4 (T) in the peripheral blood derived MNCs from mice treated with or without Saracatinib. n = 8; Mann-Whitney test: ns not significant p > 0.05.

Alteration of SDF-1α levels in the BM plasma could have important clinical implications as it is known to affect HSC function. Therefore, we next tested if systemic SRC-inhibition *in vivo*, had similar effect on *Sdf-1α* expression in the BM. Young adult mice were administered with Saracatinib (25 mg/kg body wt) thrice by oral gavage on alternate days (Figure 3A). We did not observe any gross changes in the health status of the Saracatinib treated animals; with no change in body weight (Figure S3A) or BM cellularity (Figure S3B). We then examined the expression of *Sdf-1α* in BM niche cells by performing qRT-PCR. BM derived lin^−^CD45^−^ cells were magnetically sorted and used to examine *Sdf-1α* expression (Figure 3B). We observed a significant increase in the transcript levels of *Sdf-1α*. We then performed ELISA to examine if the increase in transcript level of *Sdf-1α*, in the BM niche cells, effectively changed its protein level in BM plasma. A significant increase in the plasma levels of Sdf-1α was noted in the animals treated with Saracatinib (Figure 3C; 1.93 ± 0.21-fold increase, n = 12, p = 0.0002). Next, we addressed if there was any change in the hematopoietic function owing to elevated levels of SDF-1α in the BM plasma. First, we performed cell cycle analysis of the hematopoietic progenitor population (LSK cells) in adult BM using 7-AAD and Ki-67 staining (Figure S3C). Flow cytometry analysis was performed to examine the proportion of LSK cells in various stages of cell cycle. We noted a significantly lower proportion of LSK cells in dormant stage following an increase in SDF-1α levels (Figure 3D; 43.5 ± 7.38% in Saracatinib treated mice compared to 76.1 ± 6.05% in control animals). Owing to faster progression of cell cycle, a higher proportion of LSK cells was found to be in G1 (Figure 3E; from 10.69 ± 3.91% to 28.13 ± 5.63%), as well as SG2/M phase of the cell cycle (Figure 3F; from 13.216 ± 3.024% to 28.37 ± 4.24%), in Saracatinib treated mice, than the controls. We extended these observations to examine the effect of Saracatinib infusion on the cell cycle status of primitive HSCs. CD150^+^CD48^−^ fraction of LSK population (de Graaf et al., 2016) was examined for Ki67 expression in combination with DAPI staining (Figure 3G). The results observed for the cell cycle status of HSCs were similar to the observations made for LSK population. A decrease in the fraction of HSCs in G0 (Figure 3H), without a significant change in cells in G1 stage (Figure 3I) was noted for HSCs from Saracatinib treated mice. However, a clear increase in the fraction of HSCs in S/G2M stage of cell cycle (Figure 3J) was observed, confirming the increase in proliferation status.

After confirming that significantly higher proportion of LSK cells were actively proliferating in the BM following the inhibition of Src phosphorylation, we examined whether it resulted in any change in the HSC and HSPC populations. Flow cytometry analysis was performed on the total BM cells, to compare the frequencies of HSPC sub-populations (Figure 3K). We did not observe any change in the frequency of lin^-^c-kit^+^ (LK cells; Figure S3D) or the LSK cells (Figure S3E) following Saracatinib treatment. The LSK population was further analyzed for the expression of SLAM markers, CD150 and CD48, and frequencies of various sub-populations (CD150^+^CD48^−^ as LT-HSCs, CD150^−^CD48^−^ as ST-HSCs, CD150^+^CD48^+^ as MPP2, and CD150^−^CD48^+^ as MPP3/4) were examined as described before (de Graaf et al., 2016). The results showed an increase in the frequency of LT-HSCs (Figure 3L; 63.82 ± 5.8 to 98.26 ± 9.1 per 10^6^ BMCs), ST-HSCs (Figure 3M; 230.02 ± 30 to 308.26 ± 23.9 per 10^6^ BMCs) and MPP2 (Figure 3N; 65.02 ± 3.44 to 141.8 ± 23.6 per 10^6^ BMCs). We did not observe any change in the frequency of MPP3/4 population in the BM of Saracatinib treated mice (Figure 3O).

Ubiquitous over-expression of SDF-1α has been shown to improve G-CSF induced the mobilization of HSPCs. As our experiments showed that Saracatinib treatment led to increase in the expression of SDF-1α with concomitant increase in the phenotypic HSC population, we examined if there was any change in the circulating hematopoietic progenitors. Peripheral blood MNCs were used in methylcellulose-based colony assay and flow cytometry analysis for quantifying HSC populations (Figures 3P-3T and S3F). We did not observe any change in the frequency of CFU-Cs (Figure 3P) or phenotypic HSC populations examined, LT-HSCs (Figure 3Q), ST-HSCs (Figure 3R), MPP2 (Figure 3S) and MPP3/4 (Figure 3T).

### Systemic SRC-inhibition does not alter hematopoietic lineage commitment

**Figure 4:**
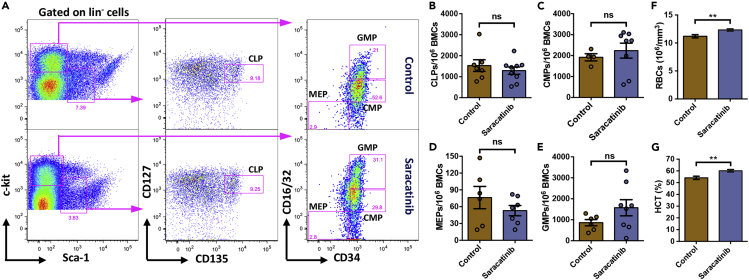
No alteration in lineage committed hematopoietic progenitors following Saracatinib treatment (A) Flow cytometry analysis of the BM MNCs for comparing the frequency of lymphoid and myeloid progenitor cells in vehicle and Saracatinib treated mice. BM MNCs gated on lin^−^ cells were analyzed for c-kit and Sca-1 expression. The lin^-^c-Kit^lo^Sca-1^lo^ cells were further gated on CD127^lo^CD135^+^ cells to identify CLPs (middle panel). Similarly, the lin^-^c-Kit^+^Sca-1^-^ cells were analyzed for the expression of CD16/32 and CD34 to identify MEPs, GMPs and CMPs (right panel). (B-E) Frequency of CLPs (B), CMPs (C), MEPs (D), and GMPs (E) in the BM of the mice treated with or without Saracatinib. Flow cytometry-based experiments were performed on BM MNCs, and quantifications were performed based on gates shown in adjacent panels (A). n = 6-7 mice, ns is not significant with p > 0.05. (F-G) Peripheral blood cell counts from vehicle (Control) and Saracatinib treated mice. The two groups of mice were compared for the number of RBCs (F), and hematocrit (HCT; G). n = 11, Mann-Whitney test: ∗∗p < 0.01.

After observing that there was an overall expansion of phenotypic HSCs in mice treated with Saracatinib, we next tested if there was any change in the hematopoietic lineage commitment. To this end, we first analyzed the frequencies of lineage committed progenitors in the BM. Common lymphoid progenitors (CLP) were identified as interleukin-7 receptor (IL7R)-α^low^ (CD127^lo^) and CD135 expressing lin^-^c-kit^lo^Sca-1^lo^ cells (Figure 4A). We did not observe any change in the frequency of adult BM CLPs upon Saracatinib treatment (Figure 4B). We then assessed if the myeloid progenitor populations were affected owing to the modulation of integrin signaling. From the total BM, lin^-^c-kit^+^Sca-1^-^ population was further analyzed for the expression of CD34 and Fcγ receptor (FcγR or CD16/32; Figure 4A). The granulocyte-monocyte progenitor (GMP), common myeloid progenitor (CMP) and megakaryocyte/erythroid progenitor (MEP) populations were identified as CD34^+^FcγR^hi^, CD34^+^FcγR^lo^ and CD34^−^FcγR^-^ cells, respectively (Figure 4A). We did not observe any increase in the CMP (Figure 4C) and MEP (Figure 4D) populations but we did observe an increase in the GMP population (Figure 4E). We also tested if the hematopoietic-lineage committed cell populations in the BM showed any change upon Saracatinib treatment (Figures S4A-S4E). Granulocyte (Gr-1^+^; Figures S4A and S4B), macrophage (F4/80^+^; Figures S4A and S4C), T cell (CD4/CD8^+^; Figures S4A and S4D) and B-cell (B220^+^; Figures S4A and S4E) populations within the BM MNCs were examined. As seen for the lymphoid and myeloid progenitor populations, we did not observe any change in the terminally differentiated blood cell populations.

In follow-up of our observations that there was no change in the frequency of lineage committed progenitors in response to Saracatinib treatment, we tested if there was any change in overall blood cell production, following the inhibition of integrin signaling. Hematology analysis was performed on the peripheral blood from control and Saracatinib treated mice (Figures 4F, 4G, S4F and S4I). Results showed that there was no change in the total white blood cell (WBC; Figure S4G) population or the proportion of lymphocytes (Figure S4G). We also did not observe any change in platelet numbers (Figure S4H). However, we observed an enhanced erythropoietic activity, reflected in increased red blood cell numbers (Figure 4F) and hematocrit (Figure 4G). Hemoglobin (Hb) level remained unaltered following the inhibition of integrin signaling (Figure S4I).

### Direct short-term exposure of HSPCs to saracatinib does not alter their proliferation status

**Figure 5:**
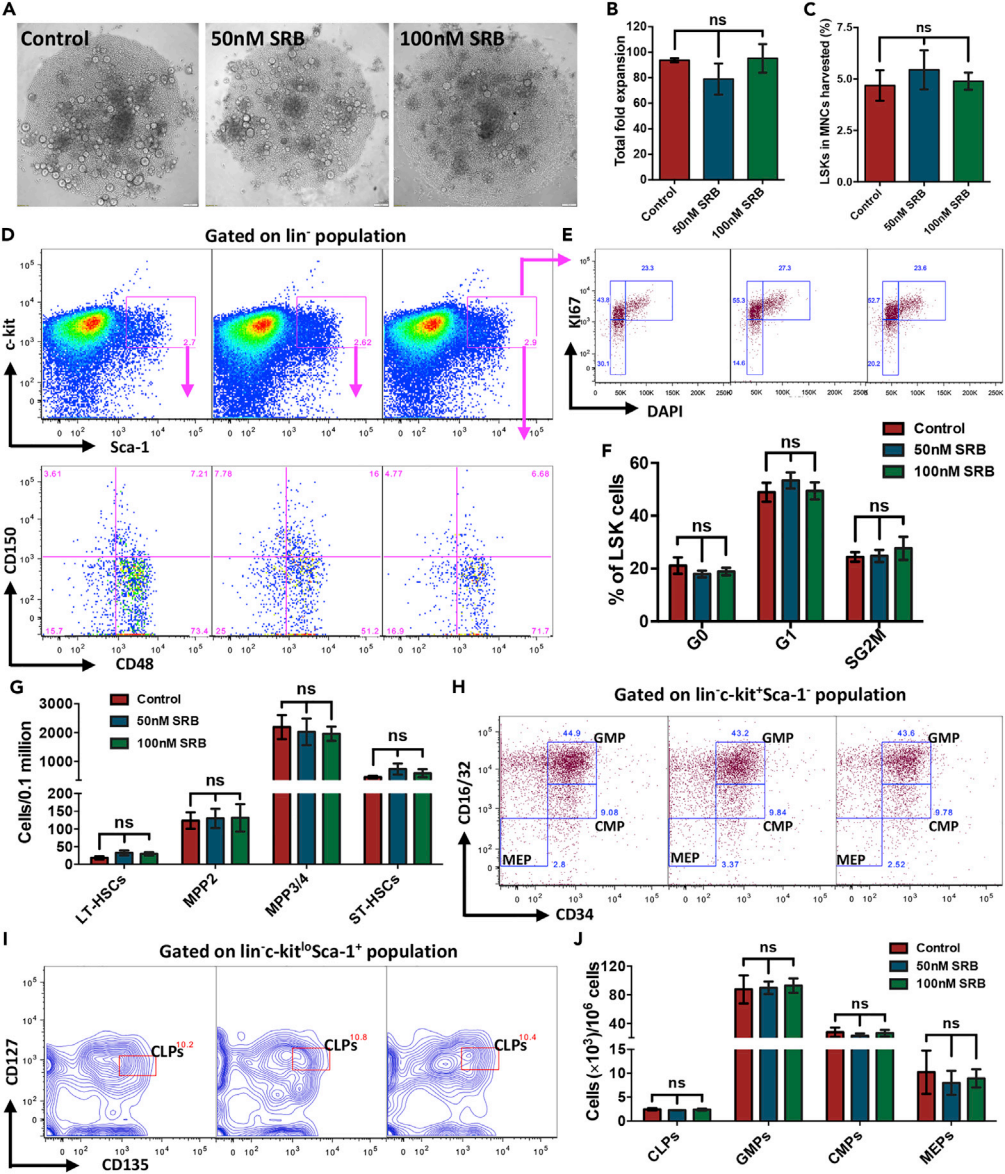
Saracatinib treatment has no direct impact on HSCs *ex vivo* (A) Phase contrast images of the clusters of progeny from sorted LSK cells cultured in serum-free medium with SCF and TPO with or without Saracatinib (SRB; 50 and 100nM) after 5 days. (B) Expansion in the total number of cells after 5 days of culture. Sorted LSK cells were cultured with or without Saracatinib and harvested cells were counted using hemocytometer. *n* = 5, ns indicates not significant with p > 0.05, Student’s *t* test. (C) Comparison of LSK cell frequency in the cells harvested after 5 days of culture with or without Saracatinib. Harvested cells were used to perform flow cytometry-based phenotyping of cells and frequency of LSK cells was examined and compared between the samples. *n* = 5, ns indicates not significant with p > 0.05, Student’s *t* test. (D) Flowcytometry plots for analysis of the cell cycle stages of LSK cells from the harvested progeny of cultured hematopoietic progenitors. The lin^−^ cells gated in the MNC population were analyzed for Sca-1 and c-kit expression to detect LSK cells. The LSK cells were further analyzed for CD48 and CD150 expression to detect LT-HSCs (CD48^−^CD150^+^ cells; upper left), ST-HSCs (CD48^−^CD150^-^ cells; bottom left), MPP2 (CD48^+^CD150^+^ cells; upper right), and MPP3/4 (CD48^+^CD150^-^ cells; bottom right). (E) The LSK cells identified in upper panel E, were further analyzed for DAPI and Ki67 staining to quantify the proportion of cells in G_0_, G_1_, and S-G_2_/M stages of cell cycle. (F) Comparison of the proportion of HSPCs (LSK cells) in various stages of cell cycle. The cells harvested after 5 days of culture with or without Saracatinib (50 and 100nM) were used for flow cytometry-based detection of various cell cycle stages based on Ki67 and DAPI staining. n = 6, ns indicates not significant with p > 0.05 using Student’s *t* test. (G) Frequency of LT-HSCs, ST-HSCs, MPP2s, and MPP3/4s in the cells harvested after 5 days of culture of LSK cells with or without Saracatinib. Flow cytometry-based experiments were performed on the harvested cells, and quantifications were performed based on gates shown in D lower panel. n = 5, ns indicates data not significant with p > 0.05, Student’s *t* test. (H) Flow cytometry analysis of the progeny of LSK cells cultured for 5 days with or without Saracatinib. To identify the myeloid progenitors MNCs gated on lin^−^ cells were first analyzed for c-kit and Sca-1 expression. The lin^-^c-Kit^+^Sca-1^-^ cells were analyzed for the expression of CD16/32 and CD34 to identify MEPs, GMPs and CMPs. (I) The lymphoid progenitor cells (CLPs) in the progeny of LSK cells after 5 days of culture was identified as CD127^lo^CD135^+^ cells within the lin^-^c-Kit^lo^Sca-1^+^ population. (J) Frequency of CLPs, GMPs, CMPs, and MEPs (D) in the cells harvested after 5 days of culture started with sorted BM derived LSK cells with or without Saracatinib (0, 50 and 100nM). Flow cytometry-based experiments were performed on the harvested cells, and quantifications were performed based on gates shown in panels H and I. n = 6-7 mice, ns indicates data not significant with p > 0.05, Student’s *t* test.

Thus far, our experiments using the systemic treatment of Saracatinib showed some differences in the hematopoietic system. In order to examine if Saracatinib had any direct on HSPC population, we sorted LSK population and performed 5-day serum-free cultures with SCF and TPO with/without Saracatinib (upto 100nM). We did not see overall expansion (Figures 5A and 5B) in the number of progenies of the LSK cells (100 cells per well plated in 96 well, round bottom plates). We then examined if there was an overall expansion of LSK cells, and did not observe any change in the expansion of LSK numbers following Saracatinib treatment (Figure 5C). This was also reflected in the cell cycle status of the LSK cells, analyzed by labeling the harvested cells with DAPI along with antibodies against Ki-67, lineage markers, Sca-1 and c-kit (Figures 5D and 5E). Following Saracatinib treatment, we did not observe any change in the proportion of LSK cells that were in G0, G1 or SG2M stages of cell cycle (Figure 5F). In addition, we also performed detailed analysis of various sub-populations (LT-HSCs, ST-HSCs, MPP2 and MPP3/4) within LSK cells based on SLAM marker expression (Figure 5D, lower panel). We did not notice any change in the frequency of these populations following Saracatinib treatment (Figure 5G). Our experiments to examine the expression of CXCR4, the receptor with specific binding affinity for SDF-1α, did not present any change (Figure S5A). We also examined if there was any change in the frequency of lineage-committed progenitor population. The CLPs (Figures S5B, 5H and 5J), GMPs, CMPs and MEPs (Figures S5C, 5I and 5J) were identified as described in our earlier experiments. The frequency of these populations was compared between the cells harvested following the 5-day culture of LSK cells with or without Saracatinib. We did not notice any change in the frequency of any of these populations. Hence, a temporally equivalent direct exposure of of Saracatinib directly on HSPC population *ex vivo*, did not have any significant effect on either the cell cycle progression or the expansion of overall HSPC population.

### Unaltered engraftment potential of hematopoietic stem cells following Src inhibition

**Figure 6:**
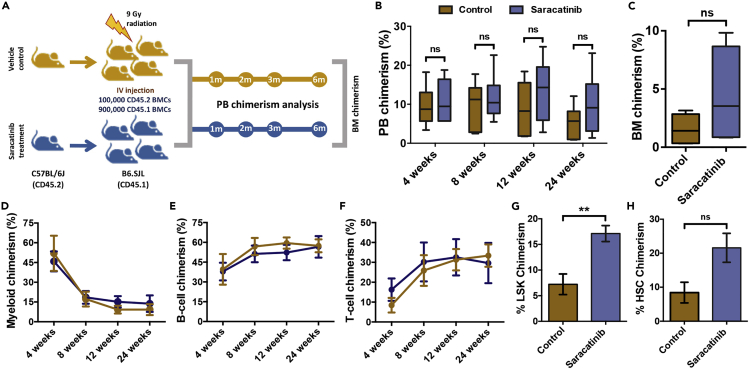
No significant effect of Saracatinib treatment on the long-term engraftment of BM cells (A) Schematic representation of long-term competitive repopulation studies. The function of HSPC population from vehicle or Saracatinib treated mice (CD45.2) was tested by transplanting in lethally irradiated mice (CD45.1). PB chimerism as well as multi-lineage chimerism was analyzed for 6-7 months. (B) Donor-derived peripheral blood (PB) engraftment in CD45.1 recipient mice treated with or without Saracatinib. Analysis was performed around 4, 8, 12 and 24 weeks’ post-transplantation. No significant change was observed. (C) Donor-derived chimerism in recipient BM, analyzed after 24 weeks of transplantation. Flow cytometry-based detection of CD45.2^+^ donor and CD45.1^+^ recipient cells was performed to compare the % chimerism in mice transplanted with BM cells from control and Saracatinib treated mice. (D-F) Multi-lineage engraftment analysis of the donor cells from control in comparison to Saracatinib treated mice. The proportion of donor-derived CD45.2^+^CD45.1^−^ cells in the total CD45^+^ PB cells from the two groups of recipient mice were further analyzed for the fraction of myeloid (CD11b/Gr-1^+^ cells; D), B-cells (B220^+^ cells; E) and T-cells (CD4/CD8^+^ cells; F). Analysis was performed after 4, 8, 12 and 24 weeks of transplantation. (G and H) Donor chimerism in LSK (G) and HSC populations (H) in recipient BM. Mice were sacrificed six months after transplantation, and the BM mononuclear cells were analyzed for the proportion of donor-derived CD45.2^+^CD45.1^−^ cells in LSK and HSC populations. Data for long-term repopulation and multi-lineage engraftment experiments are shown as mean ± SEM n*=*3, *N=*8-9; ∗∗p < 0.01 by Mann-Whitney test.

Our experiments showed larger HSC populations in Saracatinib treated mice, with a higher frequency of phenotypic LT-HSCs, ST-HSCs and MPP2s. To assess the functional relevance of these observations, we performed *in vivo* competitive repopulation assays (Figure 6A). Total BM cells were harvested for transplantation from donor CD45.2 mice, treated with or without Saracatinib. One hundred thousand donor cells along with 900,000 supporting CD45.1 BM cells were transplanted a day after the irradiation of recipient CD45.1 mice with lethal dose (9Gy). For engraftment potential, donor derived chimerism and multi-lineage engraftment was analyzed every 4 weeks, up to 12 weeks. Results showed no difference in the donor derived short-term (ST-; upto 12 weeks of transplantation) or long-term (LT-; 6-7 months) engraftment in peripheral blood of the recipient mice (Figure 6B). After 24 weeks of transplantation, there was no significant change in donor derived chimerism, in the BM of the recipient mice transplanted with BM cells from saracatinib treated mice (Figure 6C). We also analyzed multi-lineage engraftment from the donor cells and did not observe any difference in myeloid (CD11b/Gr-1^+^ cells; Figure 6D), B-cells (B220^+^ cells; Figure 6E) and T cell (CD4/CD8^+^ cells; Figure 6F) lineage differentiation. These results were unexpected as phenotypic profiling of the BM cells by flow cytometry showed increased frequency of the HSC population. Therefore, we tested if there was any increase in apoptosis within the BM HSC population in the Saracatinib treated mice. We performed Annexin V and DAPI staining, along with staining for HSC markers (Figure S6A), to examine the proportion of the apoptotic (AnnexinV^+^DAPI^−^, Figure S6B) and necrotic cells (AnnexinV^+^DAPI^+^, Figure S6C). We did not find any change in the proportion of these cells within the HSC population. Therefore, we attributed the differences in phenotypic and functional characterization of HSCs to the short-term exposure to a modulated niche. Interesting observations, however, were made when we attempted to quantify the proportion of donor-derived LSK cells and HSCs in moribund recipient mice (six months after transplantation). Surprisingly, we did observe higher levels of donor-derived chimerism in LSK (Figure 6G), but not in the HSC population (Figure 6H).

### Inhibition of integrin signaling promotes hematopoietic stem cell homing in BM niche

**Figure 7:**
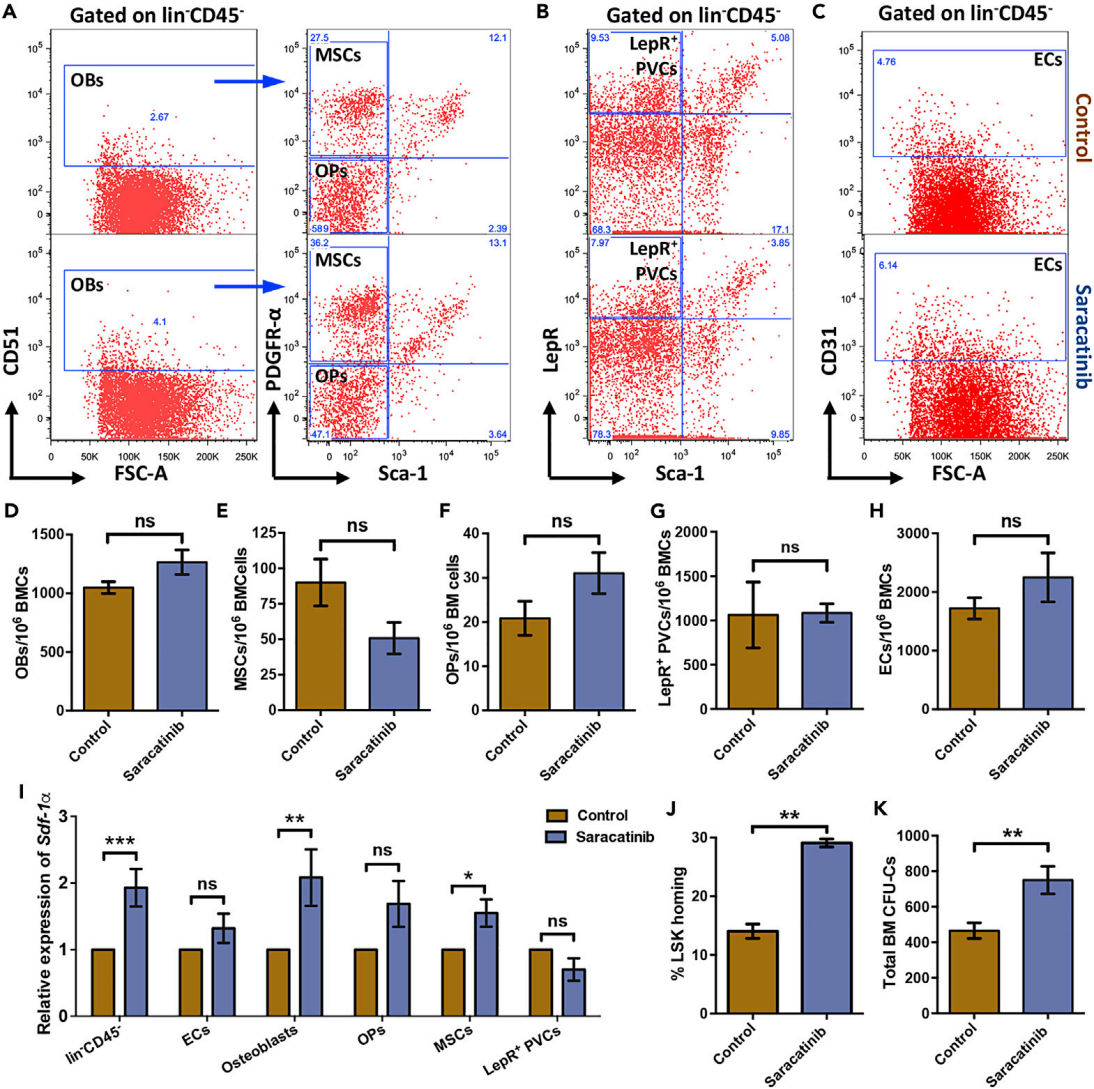
Increased LSK homing in BM following the inhibition of integrin signaling (A) Flow cytometry-based analysis and sorting of the cellular components of HSC niche in the BM from mice treated with vehicle or Saracatinib. Immuno-staining was performed using fluorophore conjugated antibodies against mouse lineage marker, CD45, CD51, Sca-1, and PDGFR-α. Lin^−^CD45^−^ BMCs (total non-hematopoietic cells) were further gated for CD51^+^ cells to identify cells of the osteoblastic lineage (OBs; lin^−^CD45^−^CD51^+^ cells). These cells were also analyzed further for the expression of Sca-1, PDGFR-α to identify the mesenchymal stromal cells (MSCs; lin^−^CD45^−^CD51^+^PDGFR-α^+^Sca-1^-^ cells) and osteoblast progenitor cells (OPs; lin^−^CD45^−^CD51^+^PDGFR-α^-^Sca-1^-^ cells). (B) To identify the perivascular cells (PVCs), the gated lin^−^CD45^−^ cells were further analyzed for the expression of LepR and Sca-1. The PVCs were identified as LepR^+^Sca-1^-^ cells. (C) The vascular endothelial fraction of the niche cells was identified as CD31^+^ cells within the gated lin^−^CD45^−^ cells. (D–H) Comparison of the frequency (per million) of OBs (D), MSCs (E), Ops (F), PVCs (G), and the vascular (H) in the BM MNCs of vehicle and Saracatinib treated mice. n = 5-7, ns indicates not significant with p > 0.05 by Mann-Whitney test. (I) Relative expression of *Sdf-1α* in the FACS sorted BM niche cells from mice treated with or without Saracatinib. Transcript levels in lin^−^CD45^−^ (total non-hematopoietic fraction), ECs, OBs, OPs, MSCs and LepR^+^ PVCs were tested by performing quantitative RT-PCR. n*=*4; p < 0.05, ∗∗p < 0.01, ∗∗∗p < 0.001, ns indicates not significant with p > 0.05 by Mann-Whitney test. (J) *In vivo* homing assays performed to examine the potential of BM niche to support incoming transplanted HSPCs following Saracatinib treatment. The proportion of LSK cells transplanted that homed in the recipient BM within 16h were plotted. *n=*5; t test: ∗∗p < 0.01. (K) Comparison of vehicle and Saracatinib treated mouse HSPCs for chemo-resistance. Methylcellulose-based colony assay is performed on the total BM cells from mice that received either vehicle or Saracatinib followed by 5-FU treatment. After the myeloablative treatment, the number of HSPCs was compared between the two groups using methylcellulose-based colony assay. Numbers of CFU-Cs in whole mouse BM is plotted for the two groups of mice. Mean ± SEM of N = 5 mice; t test: ∗∗p < 0.01.

Inhibition of integrin signaling led to increased expression of SDF-1α and expansion of phenotypic HSC population. However, no change in short- or long-term engraftment was seen in competitive repopulation assays using total BM cells. It has been shown that loss of *Sdf-1α* expression in different BM niche cell types can have divergent effects on HSC function (Ding and Morrison, 2013). This prompted us to examine if there was a particular cell type within the BM niche wherein *Sdf-1α* was differentially affected upon Saracatinib treatment. For these experiments we FACS sorted total niche cells (lin^−^CD45^−^), osteoblast population (OBs; lin^−^CD45^−^CD51^+^; Figure 7A), MSCs (lin^−^CD45^−^CD51^+^Sca-1^−^PDGFR-α^+^; Figure 7A), osteoblast progenitors (OPs; lin^−^CD45^−^CD51^+^Sca-1^−^PDGFR-α^-^; Figure 7A), LepR^+^ perivascular cells (PVCs; lin^−^CD45^−^LepR^+^; Figure 7B), and endothelial cells (ECs; lin^−^CD45^−^CD31^+^; Figure 7C). To understand if Saracatinib treatment had any effect on the composition of HSC niche in the BM, we first examined if there was any change in the frequency of these populations. Our experiments showed that there was no change in the frequency of any of these cells examined following Saracatinib treatment (Figures 7D-7H and S7B). We then performed quantitative RT-PCR experiments to analyze the expression of *Sdf-1α* in sorted niche cell populations. As shown in the previous experiments performed with magnetically sorted lin^−^CD45^−^ cells from BM, we noted higher *Sdf-1α* expression following Saracatinib treatment (Figure 7I). Further analysis showed that within the lin^−^CD45^−^ population, the osteoblast population along with the MSCs expressed higher level of *Sdf-1α* following Saracatinib treatment. On the contrary, we did not observe any change in the *Sdf-1α* expression in sorted endothelial cells, osteoblast progenitors or perivascular cells (Figure 7I).

Next, in a key set of experiments, we aimed at examining if increased SDF-1α levels in the BM affected the homing potential of the transplanted HSPCs (Figure S7A). For these experiments, we used 5-fluorouracil (5-FU)-based myeloablation model. Three doses of 5-FU were given to mice treated with carrier or Saracatinib. These two groups of mice were injected with 2 × 10^6^ PKH-26 labeled BM cells. As Saracatinib treatment could affect hematopoietic function, additional groups of mice (control and Saracatinib treated) without the transplantation of BM cells were used to examine the HSPCs that survived following chemically induced myeloablation (Figure S7A). After 16h of transplantation, the mice were sacrificed and donor derived HSPCs were identified by flow cytometry-based analysis. PKH-26^+^ LSK cells were quantified and compared between the control and Saracatinib treated groups. The results clearly showed a significantly higher proportion of transplanted HSPCs, homed in the BM of the Saracatinib treated mice (Figure 7J; 14.05 ± 1.22 to 29.11 ± 0.69% of LSK population). We also confirmed these findings with methylcellulose colony assay-based detection of homed donor cells, and examined the proportion of HSPCs homed within 16h (data not shown). Interesting observations were made from the quantification of colony-forming unit cells (CFU-Cs) from mice that were not transplanted with donor derived BM cells. Saracatinib treated mice showed higher number of CFU-Cs, following 5-FU treatment, than control mice (Figure 7K). These results showed that HSPCs in Saracatinib treated mice were more resistant to chemotherapy.

### Saracatinib treatment improves hematopoietic recovery from radiation injury

**Figure 8:**
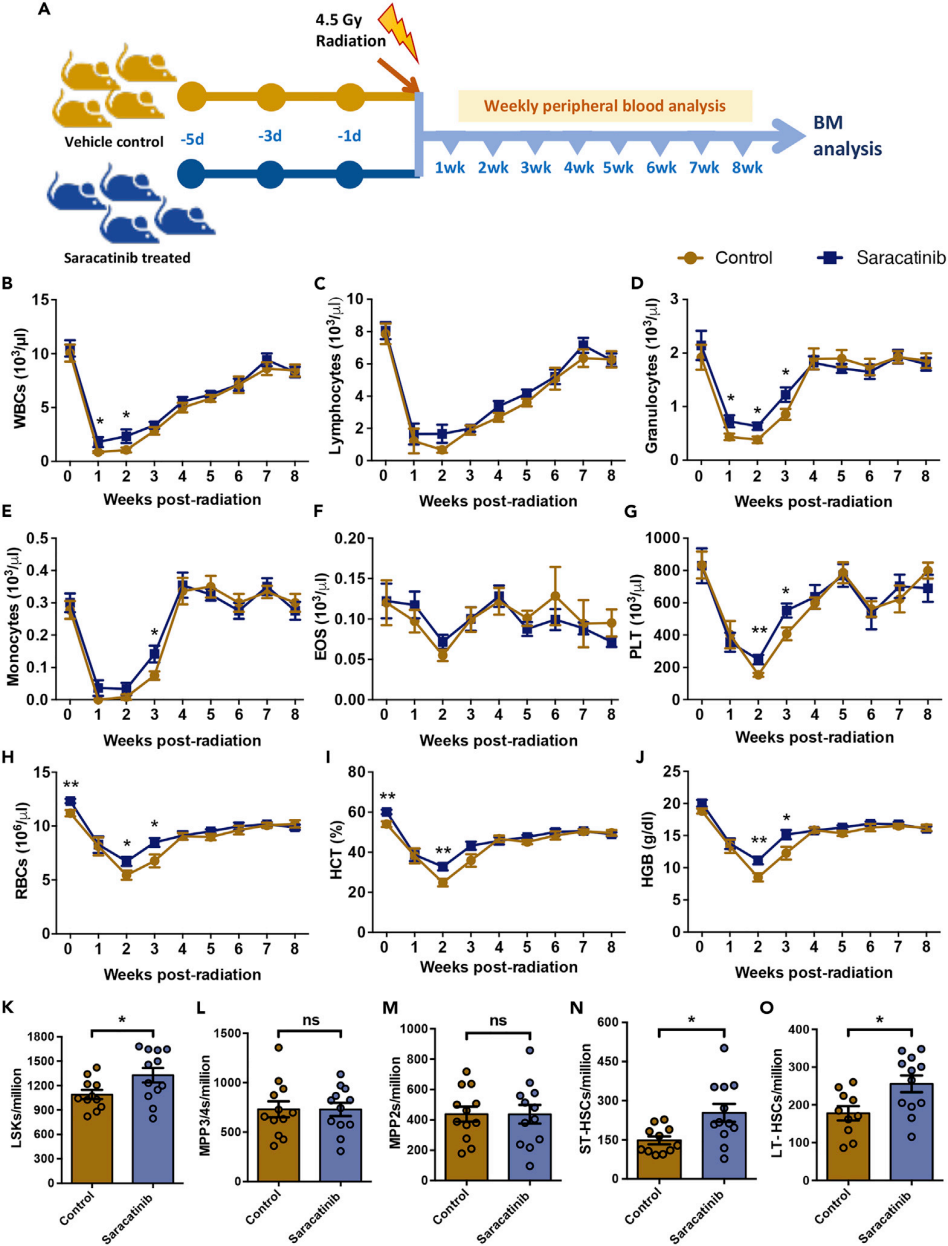
Faster recovery of hematopoietic system in mice following Saracatinib treatment (A) Schematic representation of radiation recovery experiments performed on vehicle and Saracatinib treated mice. Following sub-lethal irradiation, PB counts were counted weekly to compare the radiation recovery in the two groups of mice. After eight weeks, experiments were terminated and flow cytometry analysis was performed on BM cells. (B-J) Number of WBCs (B), lymphocytes (C), granulocytes (D), monocytes (E), eosinophils (F), platelets (G), RBCs (H), hematocrit values (HCT; I), and hemoglobin levels (HGB; J) were compared between control and Saracatinib injected groups, for a period of up to eight weeks. (K-O) Comparison of recovery of BM hematopoietic system from radiation injury in control versus Saracatinib treated mice. After 8 weeks of irradiation, the mice were sacrificed and BM mononuclear cells were analyzed for HSPC sub-populations. Frequency of LSK cells (K) and the four sub-populations, based on the expression of SLAM markers CD150 and CD48, were examined; CD150^−^CD48^+^ LSK (MPP3/4; L), CD150^+^CD48^+^ LSK (MPP2; M), CD150^−^CD48^−^ LSK (ST-HSCs; N) and CD150^−^CD48^+^ LSK (LT-HSCs; O) cells were identified and quantified. Data obtained from 10-12 independent biological replicates, was plotted as mean ± SEM ∗p < 0.05, ∗∗p < 0.01 by Mann-Whitney test.

Experiments thus far, showed that the systemic inhibition of integrin signaling led to alteration in cell cycle status and increased frequency of phenotypic HSC population. However, we did not observe any significant change in hematopoietic engraftment following the transplantation of BM cells from Saracatinib treated mice. Therefore, to understand the relevance of changes in HSPC subpopulation following Src inhibition, we performed radiation rescue experiments (Figure 8A). Mice treated with Saracatinib were compared with control mice for hematopoietic recovery following sub-lethal dose of ionizing radiation. The two groups of mice were irradiated at 4.5Gy and the recovery of the hematopoietic system was followed for 8 weeks by recording weekly blood cell counts. We observed higher white blood cell (WBC) counts in Saracatinib treated mice, one week post-irradiation, indicating a faster recovery from radiation injury (Figure 8B). There was no significant change in lymphoid cell numbers upon the inhibition of integrin signaling (Figure 8C). However, we did observe higher levels of granulocyte (Figure 8D) and monocyte (Figure 8E) populations, up to three weeks post-radiation. Furthermore, we did not observe any significant change in the kinetics of eosinophil recovery (Figure 8F), but did note higher platelet numbers in Saracatinib treated animals, two weeks after irradiation (Figure 8G). This increase could be observed for up to three weeks after inducing myeloablation. As shown in our earlier experiments (Figures 4F and 4G), we observed a significant increase in erythropoiesis in Saracatinib treated animals, even prior to radiation, reflected in RBC counts (Figure 8H) and hematocrit values (Figure 8I). This increase was maintained for upto 3-week of irradiation. Post-radiation, within two weeks, we observed an increase in the hemoglobin levels over the control group, showing a better erythropoietic function (Figure 8J). In addition to these observations, mean corpuscular volume (MCV; Figure S8A) as well as mean corpuscular hemoglobin (MCH; Figure S8B) also showed a modest increase in Saracatinib treated animals, post-radiation.

After 8 weeks of peripheral blood analysis, the animals were sacrificed and BM cells were analyzed for HSC populations. We observed an increase in the frequency of LSK population in the animals that were treated with Saracatinib (Figure 8K). Further analysis showed no change in the frequency of MPP2 (Figure 8M) and MPP3/4 (Figure 8L) sub-populations. However, a significant increase was noted in the frequency of ST-HSCs (Figure 8N) as well as LT-HSCs (Figure 8O). Hence, we observed an overall improvement in the hematopoietic repair potential of the endogenous population of BM HSPCs.

## Discussion

Extensive studies have been performed on the function of integrins that act as cell surface adhesion receptors, and are involved in a large set of cellular functions. Intracellular signal-mediated clustering of integrins and ECM binding activate diverse signal transduction pathways through the process of inside-out integrin signaling (Hynes, 1992). The vast number of cell types impacted by their function makes them one of the most important classes of cell surface molecules involved not only in cellular attachment and migration, but also in cell fate decisions (Giancotti and Ruoslahti, 1999). The short and non-enzymatic cytoplasmic domain of integrin chains interacts with diverse sets of signaling cascades, such as Ras-ERK, PI3K/AKT, and YAP/TAZ pathways, through linker and adapter proteins to affect cellular physiology (Cooper and Giancotti, 2019). However, there is no consistent information on the gene sets directly regulated by outside-in integrin signaling and it largely remains highly context dependent. Here we report the transcriptional regulation of the chemoattractant *Sdf-1α* by integrin signaling. Observations on increased Sdf-1α expression in *Postn*^*−/−*^ BM niche cells were confirmed using BM stromal cell line, ST2, via pharmacologic inhibition of Src phosphorylation. Importantly, we could confirm these findings using genetic deletion using CRISPR-Cas9 system, wherein significantly higher *Sdf-1α* expression was noted upon *Src* deletion. This increase was found to be even more pronounced than observed using pharmacologic inhibition using multiple inhibitors. These approaches confirmed the specific involvement of SRC-mediated signaling in the process. Noteworthy, deletion of Postn or Itgav was enough to induce a similar increase in the chemokine expression confirming an autocrine regulatory mechanism. These results were consistent when primary BM stroma or mouse BM derived mesenchymal stem cells (MSCs; data not shown) were subjected to SRC inhibition. We chose Saracatinib for its consistently pronounced effects on *Sdf-1α* expression. In fact, even after removal of Saracatinib from the culture medium, ST2 cells maintained higher levels of *Sdf-1α* transcripts for at least 24 h. Increased transcript levels also resulted in enhanced secretion of SDF-1α protein that reflected in increased chemo-attraction of HSPCs in a *trans*-well-based migration assay.

SDF-1α has been identified as one of the most crucial regulators of hematopoietic function. Seminal studies from the group of Tsvee Lapidot established a pivotal role of SDF-1α in homing and engraftment of transplanted HSCs (Peled et al., 1999). Blocking SDF-1α interaction with its receptor, CXCR4, led to the mobilization of BM HSCs (Aiuti et al., 1997). In fact, G-CSF-based cytokine therapy involves HSC mobilization through decrease in *Sdf-1α* expression (Petit et al., 2002). Loss of interaction between CXCR4 and SDF-1α, leads to the loss of HSC maintenance, faster cycling and exhaustion of the long-term HSC population (Tzeng et al., 2011). Contrarily, Rajendiran S et al. showed that temporal over-expression of *Sdf-1α* was enough to increase maintenance and expansion in the number of quiescent HSCs (Rajendiran et al., 2020). Strangely though, the HSCs exposed to higher levels of SDF-1α showed lower long-term multi-lineage reconstitution. Nevertheless, its expression has been used as a hallmark feature of hematopoietic niche components in the adult BM (Ding and Morrison, 2013). Through the modulation of integrin signaling, we found that it was possible to alter the level of Sdf-1α levels in BM plasma. In contrast to expectation, we observed a decrease in the quiescent HSPC population and faster cell cycle progression. However, we did observe an increase in the stem cell population without any change in the lineage committed progenitors. In this context, the involvement of a direct effect of Saracatinib-mediated inhibition of integrin signaling could not be ruled out. However, upon testing, we did not observe any change in the cell cycle progression of hematopoietic progenitors directly exposed to Saracatinib *ex vivo*. This was also reflected in unchanged frequency of HSC and the committed progenitor population within the progeny of LSK cells after a 5-day culture with Saracatinib. Unlike the committed progenitor population, mature blood cell production was affected following the inhibition of SRC signaling as we observed increased number of RBCs in peripheral blood. In comparison to the strong evidence on the effects of SDF-1α on HSC function, its role on several of the differentiated cell lineages is less well established. However, direct pro-survival effects on some of the blood cell lineages such as T-cells (Suzuki et al., 2001), and dendritic cells (Zou et al., 2001) and so forth have been demonstrated. Deletion of *Sdf-1α* led to neonatal lethality owing to the severe reduction of B-cell progenitors in fetal liver and bone marrow (Nagasawa et al., 1996). Following these studies, conditional deletion of Sdf-1α in neonatal stages showed no significant change in WBC or RBC numbers in peripheral blood (Tzeng et al., 2011). However, a direct effect of Sdf-1α elicited signaling through CXCR4 and G-protein αi has been reported on erythroid as well as myeloid progenitors (Broxmeyer et al., 2003).

Kinetics of recovery from radiation injury is an important parameter of hematopoietic function. Several reports have suggested that an improved radiation recovery indicates better function at the stem cell level (Forristal et al., 2013). However, we and others have reported that it could indicate an improved function of the hematopoietic progenitors as well (Patchen et al., 1991; Khurana et al., 2016). In our experiments, where improved frequency of HSCs as well as progenitor population was observed, following Saracatinib treatment, we noted improved hematopoietic recovery from radiation injury. This was consistent for most of the mature cell types of peripheral blood. Importantly, post-recovery from sub-lethal radiation, we found higher frequency of primitive HSCs in the BM, which clearly indicated better radiation resistance over the control group.

In one of the key experiments performed in this study, we showed an increased homing potential of transplanted HSPCs in mice treated with Saracatinib. Recipient mice were myelo-ablated chemically and the proportion of HSPCs transplanted that homed, into the BM, within 16h was quantified. As expected, owing to increased levels of SDF-1α in BM plasma, a significantly higher level of HSPC homing was noted. The role of Sdf-1α in the homing of transplanted HSCs, within the BM, has been well established. Deletion of *Sdf-1*α resulted in poorer seeding of fetal liver HSCs in developing BM and, to a lesser extent, in spleen (Nagasawa et al., 1996; Ara et al., 2003). Corroborating these findings, CXCR4, a well-known Sdf-1α receptor, was found to be a key player in the process, as its deletion showed severe defects in the establishment of BM hematopoiesis during development (Zou et al., 1998). Experiments also showed that it acts as a potent chemo-attractant for HSCs and was responsible for *trans*-endothelial migration (Imai et al., 1999). Inhibition of CXCR4-Sdf-1α results in the abrogation of homing potential of HSCs (Peled et al., 1999). On the other hand, increase in the expression of SDF-1α or CXCR4 resulted in enhanced homing of transplanted HSCs (Ponomaryov et al., 2000; Peled et al., 1999). Groups, including ours have shown that the inhibition of DPP IV that cleaves SDF-1α can efficiently improve HSC homing (Christopherson et al., 2004; Khurana et al., 2013b). Hence, the findings presented in this manuscript corroborate the role of SDF-1α in hematopoiesis, and uniquely provide a direct pathway in its transcriptional regulation. Furthermore, the results provide evidence of consistent transcriptional regulation by outside-in integrin signaling, which has hitherto been less understood.

The long-term competitive repopulation assays showed unexpected results. Against expectation, the transplantation of whole BM cells from Saracatinib treated mice that showed increased frequency of phenotypic HSCs, did not present any increase in engraftment. However, these results were consistent with previous studies using genetic model where exposure to higher levels of SDF-1α led to decreased long-term engraftment (Rajendiran et al., 2020). Surprisingly, after six months of transplantation, we could still observe increased donor chimerism in the LSK population of the recipient BM, albeit no change in donor-derived chimerism in PB. In the HSC niche of BM, the cell type targeted for *Sdf-1α* expression determines the outcome of the genetic alteration. Although the deletion of *Sdf-1α* from vascular endothelial cells and perivascular stromal cells led to the depletion of HSCs, targeting the gene in osteoblasts did not show any change in HSC population (Ding and Morrison, 2013). Therefore, we next examined the response to Saracatinib treatment in different non-hematopoietic cell populations within the BM. We observed an increase in the expression of *Sdf-1α* in the osteoblast and MSC population, but not in the vascular endothelial or perivascular stromal cells. We could not rule out the possibility that a change in the dose of Saracatinib and the duration of experiment could affect other populations and hence, would have an impact on the donor engraftment. It also remains to be tested if all these cell types respond to SRC inhibition equally, for the expression of *Sdf-1α*. A heterogeneous population of primary stroma as well as MSCs (data not shown) responded similarly. Upcoming experiments include longer-term studies on the effect of the inhibition of outside-in integrin signaling on engraftment potential of BM HSC population.

In summary, we elucidate the involvement of Src-mediated integrin signaling in the transcriptional regulation of *Sdf-1α*. Modulation of integrin signaling affected *Sdf-1α* levels in the BM plasma and resulted in improved recovery of radiation injury as well as chemo-resistance in the HSC population. In perhaps the most important experiment in this study, we show a very clear and consistent increase in the homing of transplanted HSCs in BM of the mice treated with Saracatinib. Hence, the experiments presented in this manuscript elucidate a direct role of integrin signaling in the transcriptional regulation of *Sdf-1α* that has implications on HSC function with clinical relevance.

### Limitations of the study

This manuscript unequivocally implicates SRC-mediated integrin signaling in the transcriptional regulation of *Sdf-1α* in BM niche. Clear evidence shows robust increase in homing of transplanted HSCs in Saracatinib treated mice. We observed an increase in the phenotypic HSC population in the BM, which was not reflected in the long-term multi-lineage engraftment. The underlying reason is not clear, these results could be owing to the specific set of niche cells responding to a short-term pharmacologic intervention.

## STAR★Methods

### Key resources table

**Table:** 

REAGENT or RESOURCE	SOURCE	IDENTIFIER
**Antibodies**		

anti-mouse B220 FITC	BD Biosciences	Cat#553088; Clone: RA3-6B2; RRID:AB_394618
anti-mouse Ter119 FITC	BD Pharmingen	Cat#557915; Clone: TER-119; RRID:AB_396936
anti-mouse CD11b FITC	BD Biosciences	Cat#557396; Clone: M1/70; RRID:AB_396679
anti-mouse CD3e FITC	eBiosciences	Cat#11-0031-85; Clone: 145-2C11; RRID:AB_464883
anti-mouse Gr1 FITC	BD Pharmingen	Cat#553126; Clone: RB6-8C5; RRID:AB_394642
anti-mouse CD48 FITC	eBiosciences	Cat#11-0481-85; Clone: HM48-1; RRID:AB_465078
anti-mouse Sca-1 FITC	Biolegend	Cat#122505; Clone: E13-161.7; RRID:AB_756190
anti-mouse CD16/32 APC	Biolegend	Cat#101326; Clone: 93; RRID:AB_1953273
anti-mouse CD127 APC	eBiosciences	Cat#17-1271-82; Clone: A7R34; RRID:AB_469435
anti-mouse F4/80 APC	Biolegend	Cat#123116; Clone: BM8; RRID:AB_893481
anti-mouse CD45 Biotin	Invitrogen	Cat#13-0451-85; Clone: 30-F11; RRID:AB_466447
anti-mouse Ter119 Biotin	Biolegend	Cat#116203; Clone: TER119; RRID:AB_313704
anti-mouse CD11b Biotin	BD Pharmingen	Cat#557395; Clone: M1/70; RRID:AB_2296385
anti-mouse CD3e Biotin	BD Pharmingen	Cat#553060; Clone: 145-2C11; RRID:AB_394593
anti-mouse Gr1 Biotin	BD Pharmingen	Cat#553125; Clone: RB6-885; RRID:AB_394641
anti-mouse B220 Biotin	BD Pharmingen	Cat#553086; Clone: RA3-6B2; RRID:AB_394616
anti-mouse c-kit PE	Biolegend	Cat#105808; Clone: 2B8; RRID:AB_313217
anti-mouse CD135 PE	BD Pharmingen	Cat#553842; Clone: A2F10.1; RRID:AB_395079
anti-mouse CD4 PE	eBioscience	Cat#12-0041-83; Clone: GK1.5; RRID:AB_465507
anti-mouse CD8 PE	eBioscience	Cat#12-0081-83; Clone: 53-6.7; RRID:AB_465531
anti-mouse CD34 PECy7	Biolegend	Cat#128618; Clone: HM34; RRID:AB_2721678
anti-mouse B220 PECy7	eBioscience	Cat#25-0452-82; Clone: RA3-6B2; RRID:AB_469627
anti-mouse CD150 PECY7	Biolegend	Cat#115914; Clone: TC15; RRID:AB_439797
anti-mouse c-kit APCCy7	Biolegend	Cat#105826; Clone: 2B8; RRID:AB_1626278
anti-mouse Sca-1 BB700	BD Pharmingen	Cat#742089; Clone: D7; RRID:AB_2871369
anti-mouse CD31 PECy7	eBioscience	Cat#25-0311-82; Clone: 390; RRID:AB_2716949
anti-mouse CD51 PE	BD Pharmingen	Cat#551187; Clone: RMV - 7; RRID:AB_394088
anti-mouse CD61 PE	eBioscience	Cat#12-0611-83; Clone: 2C9.G3; RRID:AB_465719
anti-mouse CD45 FITC	Biolegend	Cat#103108; Clone: 30-F11; RRID:AB_312973
anti-mouse CD48 APC	eBioscience	Cat#17-0481-82; Clone: HM48-1; RRID:AB_469408
anti-mouse PDGFRα biotin	eBioscience	Cat#13-1401-82; Clone: APA5; RRID:AB_466607
anti-mouse LepR purified	abcam	Cat#Ab5593; Clone:; RRID:AB_304969
anti-mouse CXCR4 APC	eBioscience	Cat#17-9991-80; Clone: 2B11; RRID:AB_10670877
anti-mouse c-kit APC	Biolegend	Cat#105812; Clone: 2B8; RRID:AB_313221
anti-mouse Sca-1 Super Bright 436	eBioscience	Cat#62-5981-82; Clone: D7; RRID:AB_2637287
anti-mouse Sca-1 PE-Cy7	eBioscience	Cat#25-5981-82; Clone: D7; RRID:AB_469669
anti-mouse CD150 PE	eBioscience	Cat#12-1502-82; Clone: mShad150; RRID:AB_1548765
anti-mouse CD48 eFluor® 450	eBioscience	Cat#48-0481-82; Clone: HM48-1; RRID:AB_11151336
anti-mouse Ki-67 Biotin	eBiosciences	Cat#13-5698-82; Clone: Sol A15; RRID:AB_2572794
anti-mouse Ki-67 APC	BD Biosciences	Cat#561126; Clone: B56; RRID:AB_10611874
anti-mouse SDF-1α purified	R & D Systems	Cat#MAB350; Clone: 79018; RRID:AB_2088149
anti-mouse CD45.1 Brilliant Violet 605	Biolegend	Cat#110737; Clone: A20; RRID:AB_11204076
anti-mouse CD45.2 eFluor® 450	eBioscience	Cat#48-0454-82; Clone: 104; RRID:AB_11042125
anti-CRISPR(Cas9) pur	Biolegend	Cat#844301; Clone: 7A9; RRID:AB_2565570
anti-mouse APC-Lineage antibody cocktail	BD Pharmingen	Cat#51-9003632
anti-mouse Periostin pur	R & D Systems	Cat#AF2955; RRID:AB_664123
anti-mouse CD51 pur	R & D Systems	Cat#AF1219; RRID:AB_663829
Src (32G6) Rabbit mAb	CST	Cat#2123S; RRID:AB_2106047
pSrc(Tyr416)	CST	Cat#2101S; RRID:AB_331697
β Actin (8H10D10)	CST	Cat#3700S; RRID:AB_2242334
anti-mouse AF647	Jackson ImmunoResearch	Cat#115-607-003; RRID:AB_2338931
anti-mouse Streptavidin PECy7	BD Pharmingen	Cat#557598
anti-mouse Streptavidin PerCPcy5.5	BD Pharmingen	Cat#551419
anti-rabbit AF647	Jackson ImmunoResearch	Cat#111-605-144; RRID:AB_2338078
Peroxidase anti-mouse	Jackson ImmunoResearch	Cat#715-036-151; RRID:AB_2340774
Peroxidase anti-rabbit	Jackson ImmunoResearch	Cat#111-036-144; RRID:AB_2337946
Peroxidase anti-goat	Jackson ImmunoResearch	Cat#705-036-147; RRID:AB_2340392
Annexin V FITC	BD Biosciences	Cat#556420
Phalloidin AF488	Invitrogen	Cat#A12379

**Bacterial and virus strains**		

One Shot^TM^ Stbl 3^TM^ Chemically Competent *E.coli*	Invitrogen	Cat#C737303

**Chemicals, peptides, and recombinant proteins**		

Hoechst 33342	Sigma Aldrich	B2261; CAS: 875756-97-1
DAPI	Sigma Aldrich	D9542; CAS: 28718-90-3
7AAD	BD Pharmingen	Cat# 559925
Saracatinib (AZD0530)	MedChem Express	HY-10234; CAS: 379231-04-6
DMSO	Sigma Aldrich	D8418; CAS: 67-68-5
PEG300	Merck	S7785384 005; CAS:25322-68-3
FBS	Gibco	Ref#: 10270-106
L-Glutamine	Gibco	Ref#: 25030-081
PenStrep	Gibco	Ref#: 15140-122
Stemspan SFEM II	Stemcell Technologies	Cat#09655
mTPO	Peprotech	Cat#315-14
mSCF	Peprotech	Cat#250-03
Paraformaldehyde (PFA)	Sigma Aldrich	P6148; CAS:30525-89-4
Bovine Serum Albumin (BSA)	Sigma Aldrich	A2153; CAS:9048-46-8
Tween20	Sigma Aldrich	P9416; CAS: 9005-64-5
Immobilon Western Chemiluminescent HRP Substrate	Millipore	Cat# WBKLS0100
5-Fluorouracil	Sigma Aldrich	F6627; CAS: 51-21-8
Methocult GF-M3434	Stemcell Technologies	Cat#03434
PKH-26 membrane dye	Sigma Aldrich	P9691
RPMI 1640	Gibco	Ref#:11875-093

**Critical commercial assays**		

Mouse SDF1-alpha ELISA immunoassay kit	RayBio	ELM-SDF1a
Pierce BCA Protein assay kit	Thermo Scientific	23225
RNeasy Micro RNA isolation kit	Qiagen	Cat#74034
PrimeScript 1st strand cDNA Synthesis Kit	Takara	RR037A
SYBR Green PCR kit	Takara	RR820

**Deposited data**		

Raw and analyzed data	This paper	Mendeley Data: https://doi.org/10.17632/45jtps94bd.1

**Experimental models: Cell lines**		

ST2	Dr. Catherine’s Lab, KU Leuven, Belgium	DSMZ no.: ACC 333; RRID:CVCL_2205
HEK293T	Dr. Catherine’s Lab, KU Leuven, Belgium	CRL-3216; RRID:CVCL_0063

**Experimental models: Organisms/strains**		

Mouse: C57BL/6J-CD45.2	Jackson Laboratory	RRID:IMSR_JAX:000664
Mouse: B6.SJL-PTPRCA-CD45.1	Charles River Laboratories, Raleigh, NC	Strain no. 002014
Mouse: FVB/NJ	*Centre d'Elevage* R. *Janvier,* Le Genest-St Isle, France	Strain no. 001800; RRID:IMSR_JAX:001800
Mouse: *Postn*^*−/−*^	https://doi.org/10.1038/nature10694	N/A

**Oligonucleotides**		

Primers for gRNAs used for CRISPR-Cas9 mediated knockout generation, see Table S1	This paper	N/A
Primers for oligos used for gene expression analysis, see Table S2	This paper	N/A

**Recombinant DNA**		

Lenti-Cas9-gRNA-GFP	Addgene (Depositing Lab: Jason Sheltzer)	Plasmid #124770; RRID:Addgene_124770

**Software and algorithms**		

FlowJo Version V9	FlowJo software	https://www.flowjo.com
CellProfiler 4.2.4	Cell Profiler^TM^	https://cellprofiler.org

### Resource availability

#### Lead contact

Study materials will be provided after a reasonable request. Inquiries can be directed to the lead contact, Dr. Satish Khurana (satishkhurana@iisertvm.ac.in).

#### Materials availability

This study did not generate new unique reagents. All reagents used in this study are commercially available.

### Experimental models and subject details

Six to eight weeks old C57BL/6J-CD45.2, B6.SJL-PTPRCA-CD45.1, FVB/NJ and *Postn*^*−/−*^ mice (Gender Male/Female) were bred and maintained in the animal facility at IISER Thiruvananthapuram and KU Leuven. During the experiments, mice were maintained in isolator cages at humidified constant temperature (22 ± 1°C), with a 12 h light–dark cycle. The mice were fed with autoclaved water and irradiated food, *ad libitum*. At IISER TVM, the animals were maintained as per guidelines provided by the Committee for the Purpose of Control and Supervision of Experiments on Animals (CPCSEA), Ministry of Environment and Forests, Government of India. All animal experiments were approved by the Institutional Animal Ethics Committees for the respective animal facilities.

Animals were treated with vehicle or Saracatinib via oral gavage. Three doses of 25 mg/kg Saracatinib (Cat#HY-10234; MedChemExpress LLC, NJ, USA) were given on alternate days; vehicle contained 5% DMSO, 50% PEG300 and 45% H_2_O. To perform CFU-C assays and flow cytometry based analysis of donor-derived chimerism and HSPC analysis peripheral blood (PB) was collected by tail clipping method.

### Method details

#### Bone marrow aspiration and cell sorting

Mice were sacrificed via CO_2_ asphyxiation or cervical dislocation; femurs and tibiae were harvested. Adjacent muscle tissues were removed and bones were flushed with 1X PBS using a syringe with 26G needle. The resulting cell suspension was passed through a 40μm cell strainer (Corning, USA). The filtered cell suspension was diluted with 1X PBS and centrifuged at 600xg for 10 min at 4°C. The BM MNCs were carefully resuspended in 1 mL 1X PBS and were counted manually using a Neubauer hemocytometer (Neubauer, Germany). Magnetic separation of lin^−^CD45^−^ cells was performed using anti-lineage and anti-CD45 antibodies, conjugated with magnetic microbeads (Miltenyi Biotec, Germany), as per manufacturer’s protocol.

For FACS based sorting of LSK cells, lineage depleted BM cells by magnetic separation were stained with fluorescein isothiocyanate (FITC) conjugated anti-mouse Sca-1, phycoerythrin (PE) conjugated anti-mouse c-kit, allophycocyanin (APC) conjugated anti-mouse lineage antibody cocktail (BD Pharmingen, San Diego, CA, USA). LSK cells were sorted on a BD FACS Aria III (BD Biosciences, San Jose, CA).

#### Cell culture

Mouse BM derived stromal cell line ST2 or primary stromal cells were used to examine the effect of inhibition of integrin signaling on transcriptional regulation of hematopoietic regulators. The cells were cultured with RPMI medium, supplemented with 10% FBS, 1% L-Glutamine and 1% PenStrep. After attaining 70–80% confluency, cells were treated with Src inhibitor Saracatinib (2μM) and were harvested 48h post treatment. The sorted LSK cells were cultured in round-bottom 96-well plates (Thermo fisher) in 100 μL of Stemspan (Stem Cell Technologies) supplemented with 100 ng/mL mTPO and 50 ng/mL mSCF, with or without Saracatinib (50–100nM). Cells were cultured for up to 5 days at 37°C with 5% CO_2_.

#### CRISPR-Cas9 mediated knockout generation

Three sgRNAs targeting different exonic regions of Postn, Itgav and c-SRC (Table S1) were taken from Mouse GeCKOv2 CRISPR knockout pooled library (Sanjana et al., 2014) and were cloned into the Lenti-Cas9-gRNA-GFP transfer plasmid (a gift from Jason Sheltzer; Addgene plasmid # 124770; http://n2t.net/addgene:124770; RRID: Addgene_124770) (Giuliano et al., 2019) for virus production. The annealed oligonucleotides were ligated into the linearized vector, transformed into Stbl3 cells, and sequence verified. Viral vectors were produced in HEK293T cells and the culture supernatant was used to infect ST2 cells. The transduced cells were selected by GFP-detection based FACS sorting and gene knockout were confirmed by qRT-PCR and western blotting.

#### Immunocytochemistry and imaging

ST2 cells were cultured on coverslips in a 35mm dish containing complete medium. Vehicle or Saracatinib treatment was given for 48h followed by immuno-labelling to examine cytoplasmic levels of Sdf-1α. For immuno-staining, the cells were washed with 1X PBS, followed by fixation in 4% PFA for 10 minutes at room temperature (RT) and blocking with 5% BSA and 0.01% Tween 20 solution in PBS for 1h followed. The cells were then incubated in anti-mouse SDF-1α antibodies (R&D systems, Minneapolis, MN) for 18 h at 4°C, followed by incubation with AF647 conjugated goat anti-mouse IgG1 secondary antibody, along with nuclear stain Hoechst 33342 for 2h. After mounting, the cells were imaged using an upright confocal microscope (Leica, Germany). The images were analyzed using open-source software CellProfiler^TM^.

#### Flowcytometry

Analysis of donor derived chimerism, multi-lineage engraftment, homing potential and characterization of hematopoietic system, following Saracatinib treatment, was performed by flow cytometry. For chimerism and multi-lineage engraftment, donor and recipient cells were identified as CD45.2^+^ and CD45.1^+^ cells, respectively. Within the CD45.2^+^ donor derived cells, lineage committed cells were identified as myeloid (CD11b/Gr-1^+^), T-cell (CD3e^+^) and B-cell (B220^+^) populations. eFluor450 conjugated anti-mouse CD45.2, BV605 conjugated anti-mouse CD45.1, PE conjugated Mac-1 and Gr-1, PECy7 conjugated B220, and APC conjugated anti-mouse CD3e antibodies were used (BD Pharmingen). For the characterization of HSPC sub-populations, the BM/PB derived MNCs, and the harvested progeny of cultured LSK cells were labelled with APC/FITC conjugated anti-mouse lineage antibody cocktail, PE/APC conjugated anti-mouse c-Kit, BB700/PECy7 conjugated anti-mouse Sca-1, FITC/APC-eFluor 780/eFluor 450 conjugated anti-mouse CD48 and PECy7/PE conjugated anti-mouse CD150 antibodies (ebiosciences). APC conjugated anti-mouse CXCR4 antibodies were was used to detect its expression within the HSC population. Similarly, apoptotic and necrotic cells in the HSC population were identified by using FITC conjugated anti-mouse FITC conjugated Annexin V antibodies and counter-staining with DAPI. Common lymphoid progenitors (CLPs) were detected as CD127^lo^CD135^+^ population within lin^−^Sca-1^+^c-kit^lo^ fraction of BM cells, and were detected using APC conjugated anti-mouse CD127 and PE conjugated anti-mouse CD135 antibodies, along with staining for LSK cells. Granulocyte-monocyte progenitors (GMPs; CD34^+^CD16/32^hi^ LSKs), common myeloid progenitors (CMPs; CD34^+^CD16/32^lo^ LSKs), and megakaryocyte-erythrocyte progenitors (MEPs; CD34^−^CD16/32^-^ LSKs) were identified using APC conjugated anti-mouse CD16/32 and PECy7 conjugated anti-mouse CD34 antibodies, along with labelling for identification of LSK fraction, as described above. Mature B-cells (B220^+^), T-cells (CD4/CD8^+^), macrophages (F4/80^+^) and granulocytes (Gr-1^+^) were identified using FITC conjugated anti-mouse Gr-1, APC conjugated anti-mouse F4/80, PE conjugated anti-mouse CD4/CD8, and PECy7 conjugated anti-mouse B220 antibodies. In the BM niche, endothelial cells (ECs; CD31^+^), osteoblast (OBs; CD31^−^CD51^+^), osteoblast progenitor (OPs; CD31^−^CD51^+^Sca-1^−^PDGFRα^-^ cells), mesenchymal stromal cells (MSCs; CD31^−^CD51^+^Sca-1^−^PDGFRα^+^ cells) and LepR^+^ perivascular stromal cells (PVCs; CD31^−^LepR^+^ cells) within the lin^−^CD45^−^ fraction of BM MNCs were identified using specific antibodies (details in KRT). Suitable isotype controls for each antibody were used in all experiments. The cells were analyzed by flow cytometry using FACS Aria III, FACS Canto II or Symphony (BD Biosciences, San Jose, CA).

#### Cell cycle analysis

For cell cycle analysis of HSPC population, the BM MNCs were first immuno-labelled for identification of LSK cells, as described above. After cell surface staining with anti-lineage antibody cocktail, anti-mouse Sca-1 and anti-mouse c-kit antibodies, the cells were fixed using BD Cytofix/Cytoperm buffer. The cells were then washed with Perm/Wash buffer and incubated with biotinylated Ki-67 antibody, followed by detection with streptavidin-PECy7. The cells were further washed and labelled with DAPI for 30 mins on ice. Samples were acquired on FACS ARIA III and analysed using FlowJo software.

#### *In vivo* homing assays

For homing assays, mice treated with vehicle or Saracatinib along with 5FU, were transplanted with 2 × 10^6^ CFSE-labelled bone marrow cells. The percentage of LSK cells transplanted that homed in the BM within 16h was quantified by flow cytometry, using specific antibodies mentioned before. Suitable isotype controls for each antibody were used in all experiments. The cells were analyzed by flow cytometry using FACS Aria III (BD Biosciences, San Jose, CA). Details of each antibody used in this study are provided in KRT.

#### Immuno-blotting

Cells were washed with ice cold PBS and lysed by RIPA buffer (Sigma) containing complete Mini EDTA-free protease inhibitor (Roche Molecular Biochemicals, Indianapolis, IN). Protein concentration in each cell lysate was then measured using Bicinchoninic Acid (BCA) assay kit (Thermo Scientific, Rockford, USA), following the manufacturer’s procedure. Loading dye and β-mercaptoethanol (Sigma) was added to the samples and heated at 95°C for 10 min, followed by centrifugation at 13,000xg for 10 min. In each lane of SDS-PAGE gel (12%), 25μg of protein sample was loaded, stacked at 60V for 30 min and separated at 110V for 1h. The resolved proteins were then transferred to PVDF membrane (Bio-Rad Laboratories) at 90V and 4°C overnight using a Bio-Rad Mini Trans-Blot Electrophoretic Transfer System. Membranes were blocked for 1h at room temperature with 5% non-fat milk and incubated with different primary antibodies at 4°C overnight. Membranes were washed and incubated with horseradish peroxidase (HRP)-labelled secondary antibodies for 2h at RT. Immuno-reactive bands were then visualized using Immobilon Western Chemiluminescet HRP Substrate (Cat#WBKLS0100 Millipore Corporation, Billerica, MA,USA). Imaging was performed on a Biorad chemidoc platform and level of intensities were analyzed using Bio-Rad Quantity One software.

#### ELISA

ELISA was performed to quantify the levels of Sdf-1α in culture supernatant of ST2 cells or BM plasma from mice treated with vehicle (control) or Saracatinib. Supernatant from ST2 cell cultures was collected after 48h of treatment, and stored at −80°C until used. To obtain BM plasma, hind limb bones of mice were flushed with 200μL PBS. Cells were pelleted down and the plasma was used to quantify Sdf-1α levels using the mouse ELISA immunoassay kit (ELM-SDF1a, RayBiotech, GA, USA), following the manufacturer’s instructions. ELISA was performed using 10μL of diluted sample and the plates were read on a TECAN plate reader (BioTek, USA).

#### Long-term competitive repopulation assays

For comparing the frequency and hematopoietic function of the HSCs from control and Saracatinib treated mice (both CD45.2), 100,000 freshly isolated whole BM cells were transplanted in lethally irradiated (9Gy) 8–12 weeks old female mice, fed on enrofloxacin (Baytril®) containing water, along with 900,000 WBMCs (CD45.1). Peripheral blood chimerism and multilineage engraftment analysis was performed every 4 weeks. After 6–7 months of transplantation, the mice were sacrificed and donor derived chimerism within total BM MNCs, LSK and HSC population were examined by flow cytometry.

#### Radiation rescue experiments

The mice treated with or without Saracatinib were irradiated sub-lethally (4.5Gy) using an X-ray based RAD SOURCE RS-2000 biological Irradiator (Rad Source Technologies, Alpharetta, GA). The extent of hematopoietic injury and the follow up recovery from radiation injury was monitored by PB analysis, at one-week interval, for 8 weeks. The PB samples were collected by tail clipping, in EDTA coated microcuvettes (Sarstedt) and analyzed on a scil Vet ABC animal blood counter (Horiba ABX, Montpellier, France). Peripheral blood levels of RBCs, hematocrit, hemoglobin, mean corpuscular volume (MCV), mean corpuscular hemoglobin (MCH), total WBCs, monocytes, granulocytes, platelets, eosinophils and lymphocytes were measured. Eight weeks after radiation, the mice were sacrificed and the frequency of LSK cells and HSCs was examined by flow cytometry.

#### Chemo-resistance analysis

For comparison of chemo-resistance in HSPC population of vehicle versus Saracatinib treated mice, myeloablation was chemically induced by injecting 125 mg/kg body weight of 5-FU, intraperitoneally, along with vehicle/Saracatinib treatment. Three doses of 5-FU were given to each mice (4, 3 and 1 day prior to analysis), before the mice were sacrificed. Total BM cells were used to perform hematopoietic colony-forming assays. Methocult GF-M3434 (STEMCELL Technologies Inc., Canada) was used to culture whole BM cells from mice treated or untreated with Saracatinib. Two million total BM cells were suspended in the semisolid medium thoroughly, using blunt end cannula (16G, 1.5 inches; Monoject^TM^), in 2 mL colony assay medium. One mL of the suspension was added per well, in 6-well plates, and cultured for 12 days in a humidified incubator at 37°C and 5% CO_2_. Hematopoietic colonies were identified and counted using an inverted bright field microscope.

#### *In vitro* migration and adhesion assays

*In vitro* trans-well migration assays were performed with 50,000 ST2 cells were plated per well in 24 well plates and cultured with or without Saracatinib for 48h. Lineage depleted BM MNCs were labelled with PKH-26 membrane dye, according to the manufacturer’s instructions (Sigma, St Louis, MO, USA). The cells were re-suspended at 1 × 10^7^ per mL of Diluent C. The cell suspension was mixed with an equal volume of 4 mM PKH26 dye (in Diluent C) for 5 min at room temperature. An equal volume of fetal bovine serum (FBS) was added to stop the reaction and the cells were washed with medium containing 10% FBS. 200,000 PKH-26^+^ cells in 200 μL of chemotaxis buffer (RPMI 1640, 1% fetal bovine serum [FBS; Gibco BRL, Grand Island, NY], and antibiotics) were added to the upper chamber of a 6.5 mm, 3μm pore size Transwell (Costar, Cambridge, MA). Chambers were incubated at 37°C, 5% CO_2_ for 3 hours. Cells migrating into the lower chamber were counted by flow cytometry to distinguish between the fluorescently labelled HSPCs and unlabelled ST2 cells in a non-contact based culture. The proportion of cells that migrated to the lower chamber was quantified and compared between the two treatments.

For adhesion assays, a contact based cultures were performed using PKH-26^+^ lineage depleted cells that were added to the ST2 cells cultured with or without Saracatinib. The plates were incubated for 3h at 37°C and 5% CO_2_. Non-adherent cells were removed and adherent cells were harvested along with the feeder layer. To compare adhesion potential of the HSPCs to ST2 cells cultured with or without Saracatinib, flow cytometry was performed to quantify the fluorescently labeled HSPCs that attached to ST2 cells.

#### Quantitative RT-PCR

For the analysis of level of expression of various genes, qRT-PCR was performed. Freshly isolated lin^−^CD45^−^ BM cells from; 1) *Postn*^*+/+*^ and *Postn*^*−/−*^ mice, or 2) mice treated with or without Saracatinib, were used for analyses. In other experiments, mouse BM stroma derived cell line ST2 or primary BM stromal cells were used for gene expression analysis followed by culture with or without various inhibitors. Total RNA was isolated from the cells using RNeasy Micro RNA isolation kit (Qiagen, Hilden, Germany). DNase treatment of the RNA was performed using the Turbo DNase kit (Cat# Ambion, Austin, TX, USA). The purity and concentration of RNA were assessed using a Colibri spectrophotometer (Titertek-Berthold, Germany). 100ng-1μg of RNA from each sample was used to synthesize cDNA, using PrimeScript 1st strand cDNA Synthesis Kit (Takara, Japan), according to the manufacturer’s protocol. Quantitative PCR was then carried out using SYBR Green PCR kit (Takara, Japan). The reactions were carried out on a BioRad C-1000 cycler (Bio-Rad, USA) using the following program: 2 min at 50°C, 1 min at 95°C and 40 cycles of 30 sec at 95°C and 30 sec at 60°C. The list of primers used is given in Table S2. For heatmap visualization of the relative expression of various cytokines through RT-PCR analysis, ΔCt values were row-wise Z-score scaled before visualization, with red and blue indicating higher and lower expression, respectively. R packages ggplot2 and heatmap.2x were used to generate the heatmap.

### Quantification and statistical analysis

All data are represented as mean ± SEM. Normal distribution of data was tested using the Shapiro-Wilk test. The equality of group variance was tested using Brown-Forsythe test. Comparisons between samples, from two groups with normally distributed data with equal variance, were made using the unpaired two-tailed Student’s *t-test*. Mann Whitney test was used for comparing two groups where data were non-normally distributed. Extreme outliers were identified as per the 1.5 IQR rule in Microsoft Excel and are defined as the data points that fall below Q1 − 1.5 IQR or above Q3 + 1.5 IQR. Statistical analyses were performed with Microsoft Excel or GraphPad Prism 6. For all analyses, p-values ≤0.05 were accepted as statistically significant, “n” represents the biologically independent repeats.

## Data Availability

• Raw, analyzed data and Original western blot images have been deposited at Mendeley Data and are publicly available as of the date of publication. The DOI is listed in the key resources table. • No code data was generated. • Any additional information required to reanalyse the data reported in this paper is available from the lead contact upon request.
