# Supplementary material for: Inhibition of SRC-mediated integrin signaling in bone marrow niche enhances hematopoietic stem cell function
Source: iScience. 2022 Sep 19;25(10):105171. doi: 10.1016/j.isci.2022.105171 (PMC9530850; doi:10.1016/j.isci.2022.105171)
Supplement: Document S1. Figures S1–S8, Tables S1 and S2 [file mmc1.pdf]

iScience, Volume *25*

Supplemental information

Inhibition of SRC-mediated integrin signaling in bone marrow niche enhances hematopoietic stem cell function

Irene Mariam Roy, P.V. Anu, Samantha Zaunz, Srinu Reddi, Aravind M. Giri, Rithika Saroj Sankar, Sarah Schouteden, Joerg Huelsken, Catherine M. Verfaillie, and Satish Khurana

Supplementary Information

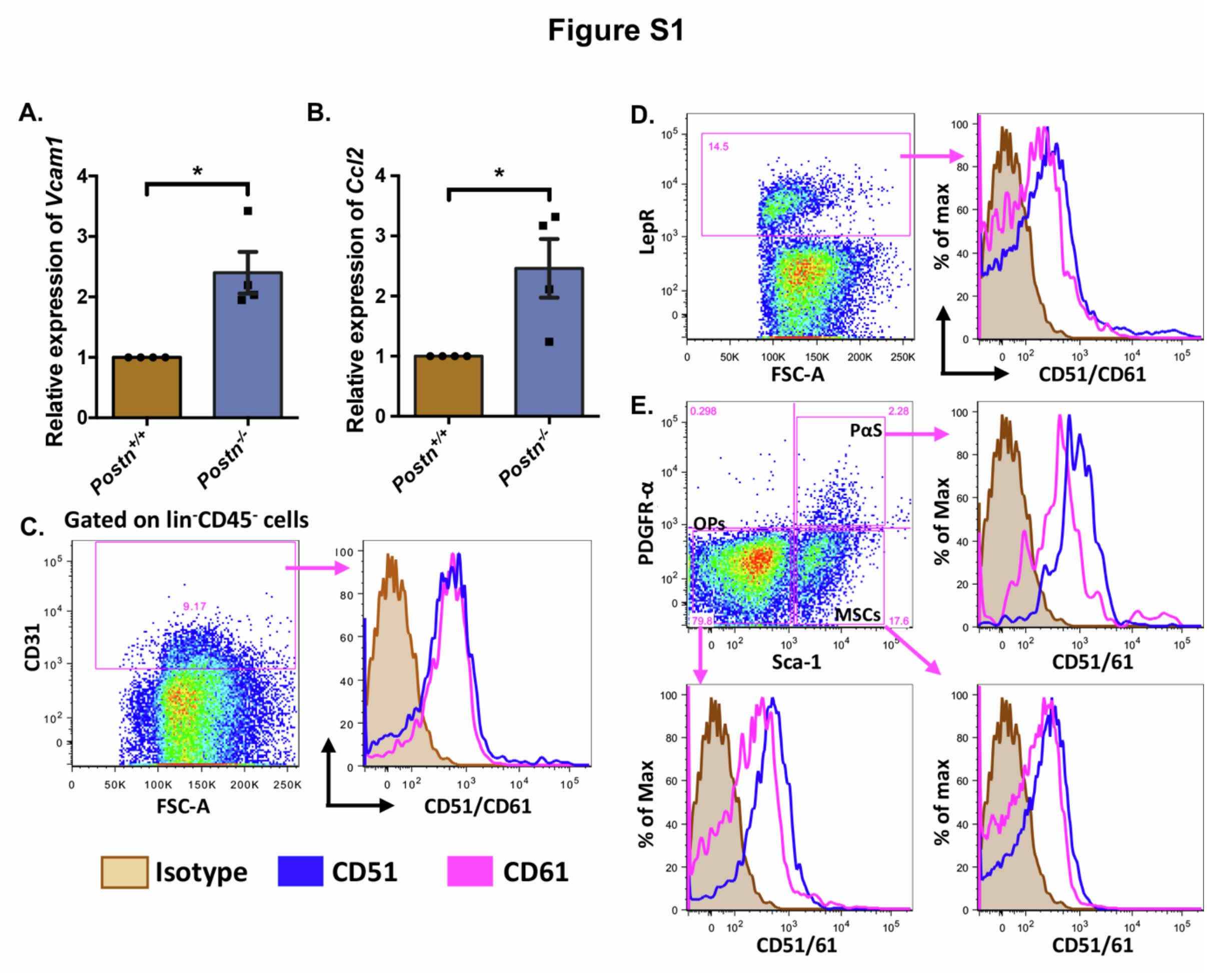

Figure S1. Inhibition of Src phosphorylation increases *Sdf-1α* in BM stromal cells, related to Figure 1.

A) Relative expression of *Vcam1* in lin$^-$CD45$^-$ BM cells from *Postn*$^{+/+}$ and *Postn*$^{-/-}$ mice by qRT-PCR. The cells were magnetically sorted from total BM MNC population and quantitative RT-PCR was performed using total RNA isolated. *n*=4; t-test: * p<0.05.

B) Quantitative RT-PCR experiments were performed to examine the expression of *Ccl2*. BM derived lin$^-$CD45$^-$ cells from *Postn*$^{+/+}$ and *Postn*$^{-/-}$ mice were used in quantitative RT-PCR experiments. *n*=4; t-test: * p<0.05.

C) Flow cytometry analysis of endothelial cell within the non-hematopoietic for the expression of ITGAV (CD51) and ITGB3 (CD61). Total BM MNCs were first gated for lin-CD45- cells (left), which were further analysed for the expression of integrin chains.

D) Analysis of LepR$^+$ perivascular cells for the expression of ITGAV and ITGB3 by flow cytometry (right). LepR$^+$ cells were gated on lin-CD45- cells within the BM MNC population (left).

E) Expression of ITGAV and ITGB3 in mesenchymal stromal cells (MSCs; PDGFRα$^-$Sca-1$^+$ cells), PαS (PDGFRα$^+$Sca-1$^+$) cells, and osteoblast progenitor (OP; (PDGFRα$^-$Sca-1$^-$) cells.

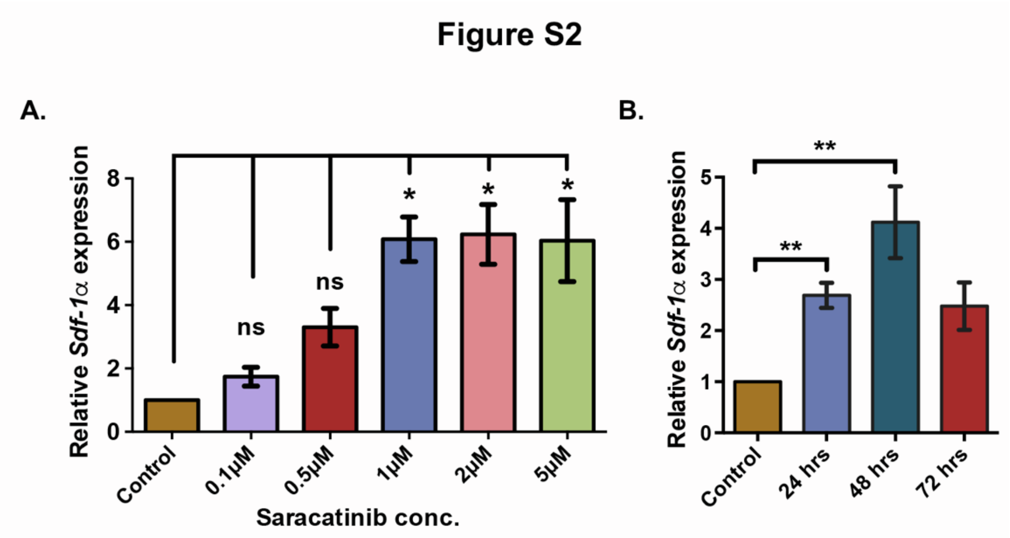

Figure S2. Dose and time response for the effect of SRC-inhibition on *Sdf-1α* expression, related to Figure 2.

A) Relative expression of *Sdf-1α* in ST2 cells treated with different concentrations of Saracatinib. Upto 5µM of Saracatinib was tested for is effect on *Sdf-1α* expression. *n*=3-5; Mann-Whitney test, ns p>0.05, * p<0.05

B) Relative *Sdf-1α* expression in ST2 stromal cells cultured with Saracatinib for different time periods. The cells were cultured with or without Saracatinib for upto 12, 24, 48 and 72 hours. Total RNA was isolated and used to performed quantitative RT=PCR to examine *Sdf-1α* transcript level. n=6, Mann-Whitney test * p<0.05, ** p<0.01

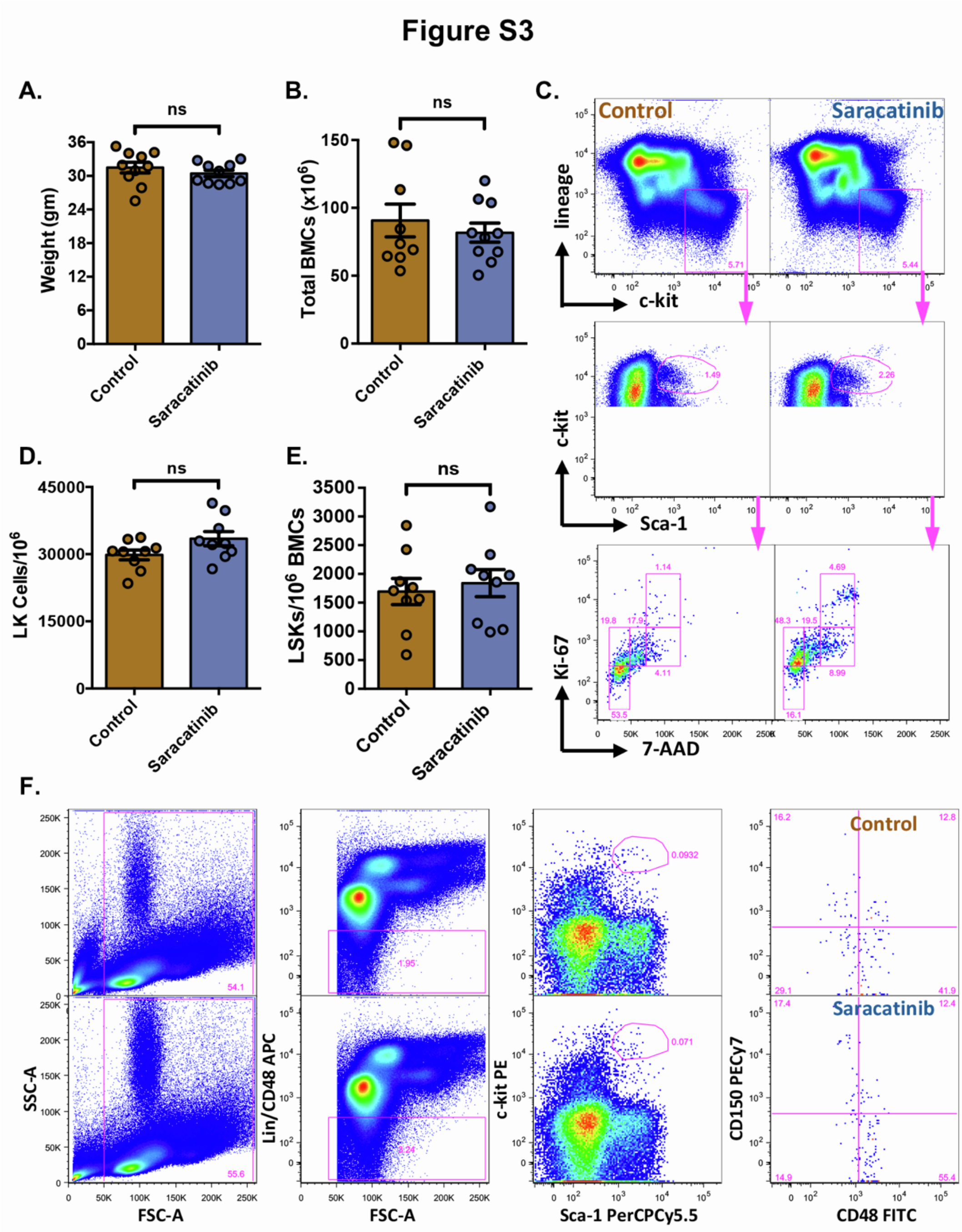

Figure S3: Treatment with SRC inhibitor Saracatinib alters cell cycle status of HSCs in vivo, related to Figure 3.

A) Total body weight of the mice treated with or without Saracatinib. Three doses of vehicle alone or Saracatinib (25mg/kg), through oral gavage, were given on alternate days. *n*=6, *N*=10; Mann-Whitney test: ns - Not significant p>0.05.

B) Comparison of the total BM MNCs from the hind limbs of mice treated with or without Saracatinib. n=9, Mann-WHitney test ns not significant p>0.05

C) Flowcytometry plots for the analysis of cell cycle stages of LSK cells from the BM MNCs of vehicle and Saracatinib treated mice. The LSK cells gated in the middle panel were further analyzed for 7-AAD and Ki67 staining to quantify the proportion of cells in G_0, G_1, S and G_2/M stages of cell cycle. (n=6)

D) Flowcytometry based analysis of $lin^-c\text{-}kit^+$ (LK) cells in the total BM MNCs from mice treated with or without Saracatinib. n=9 Mann-Whitney test: ns - Not significant $p>0.05$.

E) Comparison of the frequency of hematopoietic stem and progenitor cell population (LSK cells) in the BM of mice treated with vehicle or Saracatinib. n=9, Mann-Whitney test: ns shows not significant with $p>0.05$.

F) Flow cytometry based analysis of PB cells to quantify the frequency of various hematopoietic stem and progenitor populations in vehicle and Saracatinib treated mice. The PB lin^-CD48^+ cells gated for $Sca\text{-}1^+c\text{-}kit^+$ cells (LSK cells) were further analyzed for CD48 and CD150 expression for detecting LT-HSCs ($CD48^-CD150^+$ cells; upper left), ST-HSCs ($CD48^-CD150^-$ cells; bottom left), MPP2 ($CD48^+CD150^+$ cells; upper right), and MPP3/4 ($CD48^+CD150^-$ cells; bottom right).

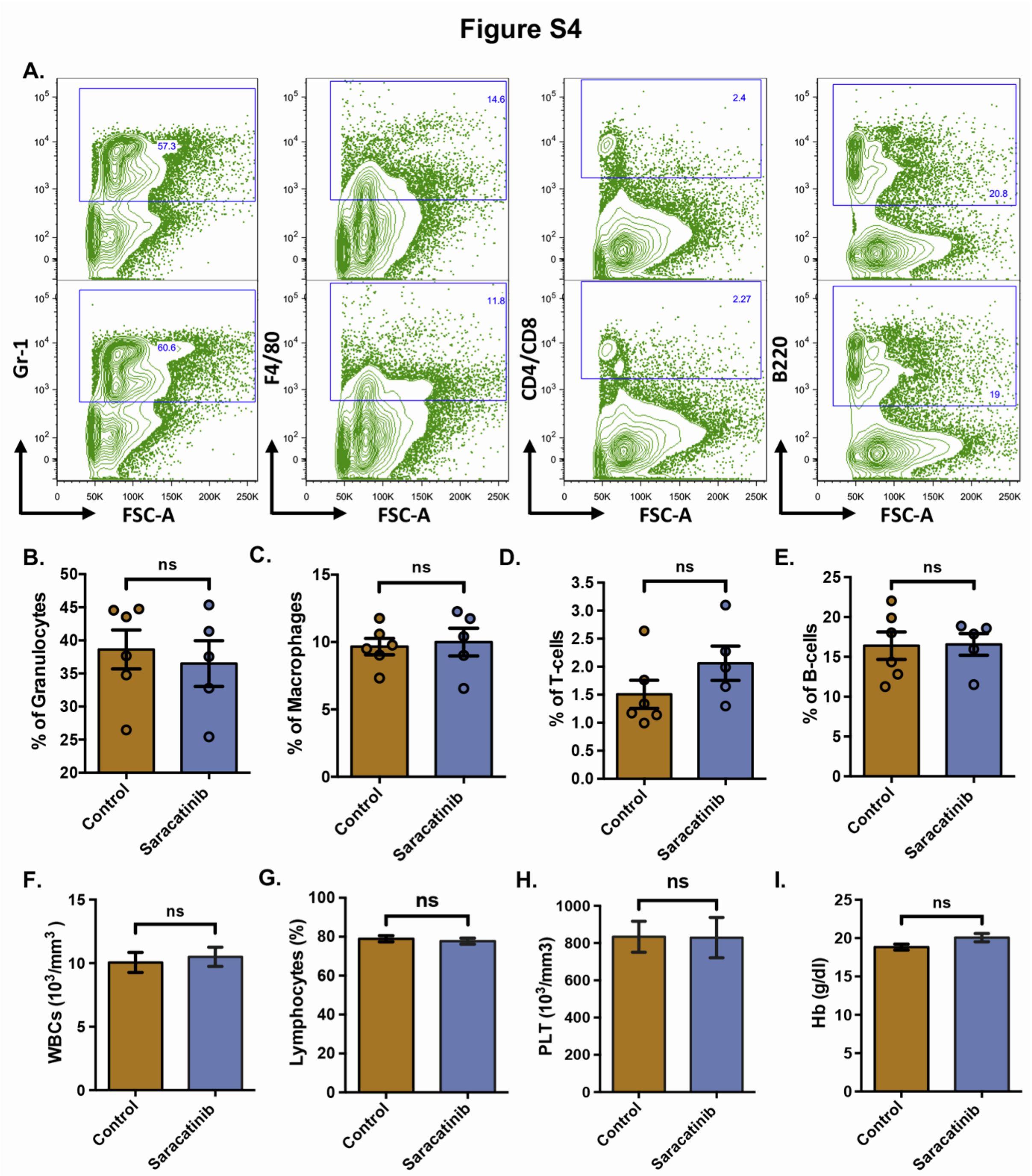

Figure S4: No alteration in lineage committed cells in BM following Saracatinib treatment, related to Figure 4.

A) Flow cytometry plots for the analysis of lineage committed mature blood cell populations. Mononuclear cells harvested from Control and Saracatinib treated mice were used for flow cytometry analysis of granulocytes (Gr-1$^+$), macrophages (F4/80$^+$), T-cells (CD4/CD8$^+$), and B-cells (B-220$^+$).

B) The proportion of Gr-1$^+$ myeloid cells in the BM cells of control and Saracatinib treated mice.

C) Comparison of BM macrophage population in control and Saracatinib treated mice.

D) T-cell (CD4/CD8$^+$) population in vehicle and Saracatinib treated mouse BM.

E) B220$^+$ B-cells compared between control and Saracatinib treated mice.

F-I) Peripheral blood cell counts from vehicle (Control) and Saracatinib treated mice. The two groups of mice were compared for the number of WBCs (F), lymphocytes (G), platelets (H), and hemoglobin (Hb; I). n=11, ns indicates not significant with $p>0.05$ Mann-Whitney test.

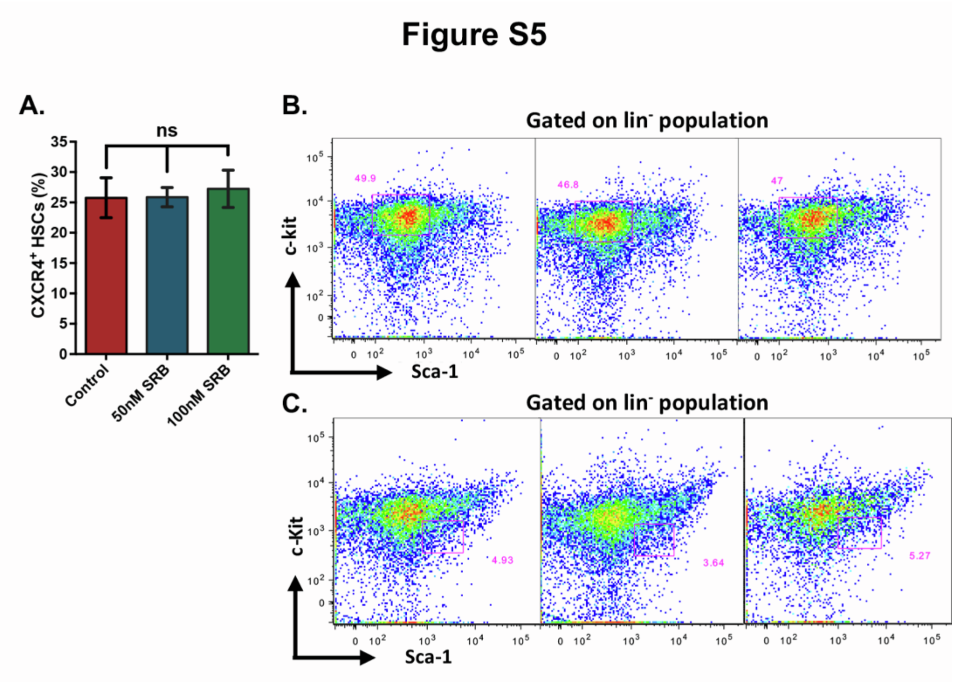

Figure S5: No significant change in the SDF-1α receptor expression on HSCs treated with Saracatinib, related to Figure 5.

A) Comparison of cell surface CXCR4 expression on the progeny of LSK cells following 5 days of culture with or without Saracatanib (50nM and 100nM). *n*=5, ns indicates not significant with p>0.05, Mann-Whitney test.

B) Flow cytometry plots for gating lin-c-kit+Sca-1- within the total MNCs harvested after culture. This population was further used for gating GMPs, CMPs and MEPs based on CD16/32 and CD34 expression Fig 5 H.

C) Flow cytometry profile of the MNCs gated on $lin^{-}c\text{-}kit^{lo}Sca\text{-}1^{+}$ cells for further analysis of CLP frequency in LSK cell progeny harvested after 5 days of culture. The gated cells were analyzed for the expression of CD127 and CD135 to identify CLPs as $CD127^{lo}CD135^{+}$ cells as shown in Fig. 5I.

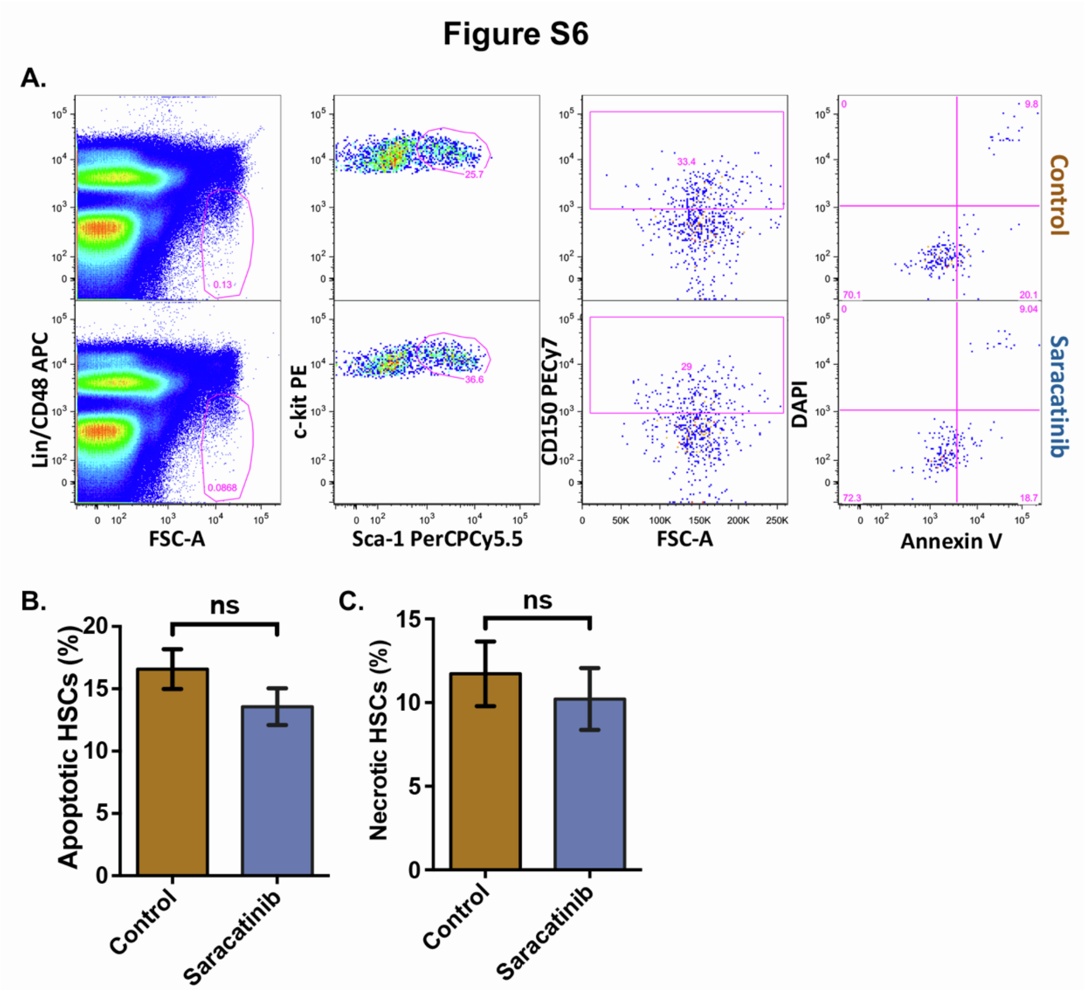

Figure S6: No significant change in the apoptotic stage of Saracatinib treated HSCs , related to Figure 6.

A) Flow cytometry plots to examine the apoptotic and necrotic HSCs in control and Saracatinib treated mice using immunolabelling for Annexin V, and DAPI staining. From within the HSC population identified as CD150+CD48- LSK cells, the necrotic (Annexin V^+DAPI^+) and apoptotic (Annexin V^+DAPI^-) cells were identified.

B) Comparison of the proportion of apoptotic HSCs after Saracatinib treatment when compared to control. n=3, ns indicates not significant with p>0.05 by Mann-Whitney test.

C) Proportion of necrotic HSCs in the BM of control and Saracatinib treated mice. n=3, ns indicates not significant with p>0.05 by Mann-Whitney test.

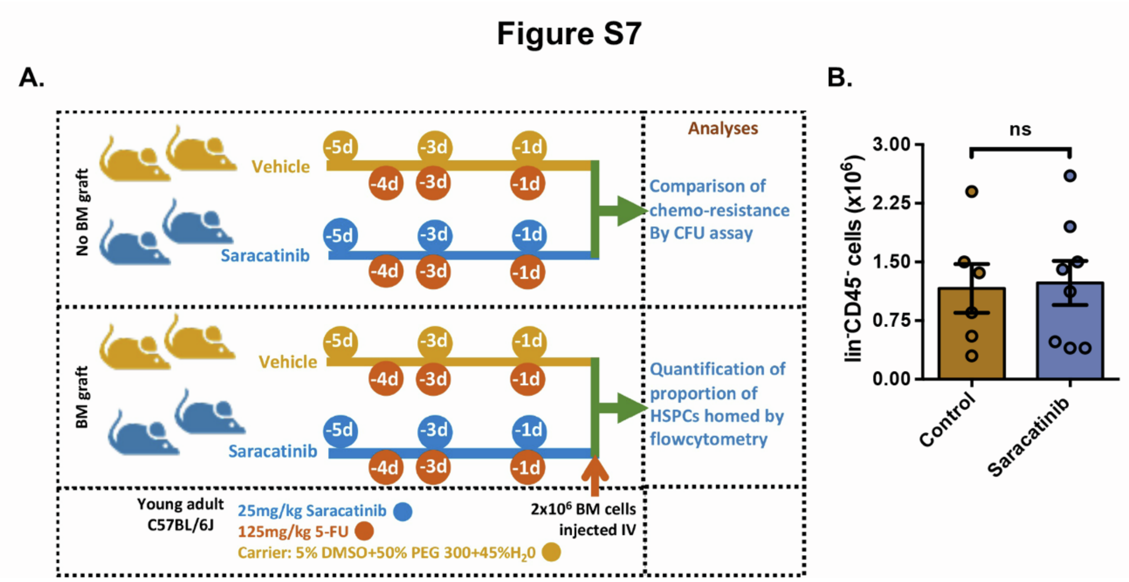

Figure S7: Radiation recovery assays to compare hematopoietic function post-Saracatinib treatment, related to Figure 7

A) Schematic representation of the methodology followed to examine the homing potential of HSPCs. The mice that received either vehicle or Saracatinib underwent 5-FU treatment for myeloablation and BM conditioning. The two groups of mice then received two million CFSE labelled total BM cells per animal. After 16h of grafting, BM cells from the recipient mice were examined to quantify the numbers of donor derived LSK fraction.

B) Comparison of the frequency of total niche population identified as lin$^-$CD45$^-$ (non-hematopoietic fraction) in control versus Saracatinib treated mice. *n*=3, *N*=6, ns indicates not significant with p>0.05 by Mann-Whitney test

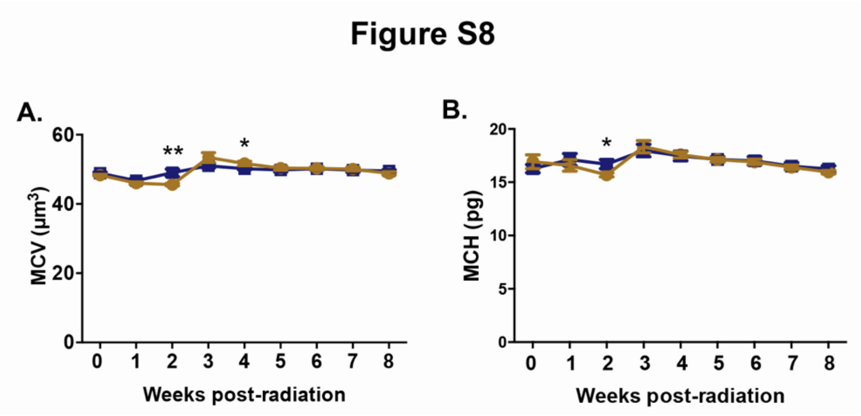

Figure S8. Faster recovery of hematopoietic system in mice following Saracatinib treatment, related to Figure 8

Radiation recovery experiments were performed on vehicle and Saracatinib treated mice. Following sub-lethal irradiation, PB counts were counted weekly to compare the radiation recovery in the two groups of mice.

A) Comparison of vehicle and Saracatinib treated mice for mean corpuscular volume (MCV) in the course of recovery from radiation injury. Weekly recorded data is plotted for the two groups of mice.

B) Mean corpuscular hemoglobin (MCH) values compared between control and Saracatinib injected groups, for a period of eight weeks.

Data obtained from 10-12 independent biological replicates, was plotted as mean ± SEM. * $p<0.05$, ** $p<0.01$ by Mann-Whitney test.

Table S1. List of gRNAs used for CRISPR-Cas9 mediated knockout generation, related to STAR Methods

Gene	gRNA	gRNA sequence	Target Exon
Postn	1	GTACTTCGCGCCGAGTAACG	4
	2	CCTGATCCCGACCCCTGATG	1
	3	TTCTCCCAAGCCTCGTTACT	4
Itgav	1	TTTAAGTCCCACCAGTGGTT	5
	2	TTATGCCAAAGATGACCCAC	5
	3	CCTGTGCTCCATTGTACCAC	6
Src	1	CTCGGCCGCGGGCGGCACGA	7
	2	CTCAACGCCGAGAACCCGAG	10
	3	CGCCGCCTTCGTGCCGCCCG	7

Table S2. List of oligos used for gene expression analysis, , related to STAR Methods

1	Mm *Actb* (F)	GATGTATGAAGGCTTTGGTC
	Mm *Actb* (R)	TGTGCACTTTTATTGGTCTC
2	Mm *Sdf-1α* (F)	GGAGAAAGCTTTAAACAAGAGGC
	Mm *Sdf-1α* (R)	GGCCCTTCCCTAACACTGAC
3	Mm *Tpo* (F)	GGAGAAAGCTTTAAACAAGAGGC
	Mm *Tpo* (R)	GGCCCTTCCCTAACACTGAC
4	Mm *Il-6* (F)	AGCCAGAGTCCTTCAGAGAGAT
	Mm *Il-6* (R)	CATAACGCACTAGGTTTGCCG
5	Mm *Ccl2* (F)	CCTGCTGTTCACAGTTGCCG
	Mm *Ccl2* (R)	CACAGACCTCTCTCTTGAGCTT
6	Mm *Vcam1* (F)	GCCACCCTCACCTTAATTGC
	Mm *Vcam1* (R)	CAGCACACGTCAGAACAACC
7	Mm *Scf* (F)	AGCCAGAGTCCTTCAGAGAGAT
	Mm *Scf* (R)	CATAACGCACTAGGTTTGCCG
8	Mm *Mmp9* (F)	TCCCCAGAGCGTCATTCGCG
	Mm *Mmp9* (R)	CACGTAGCCCACGTCGTCCAC
9	Mm *Upa* (F)	CTGGCTGGCGAGCCTGTTCC
	Mm *Upa* (R)	CAGGCAGGGCCGACCTTTGG
10	Mm *Angptl3* (F)	AAAGGGCTTTGGGAGGCTCGATG
	Mm *Angptl3* (R)	GAGGGCCCCAGGGATATTGCCA
11	Mm *Rhamm* (F)	GCTCGCCCTGGCTGAGTTGG
	Mm *Rhamm* (R)	ATGGCTTGGGCGTGAGCAGC
12	Mm *Icam1* (F)	ACCTGCACTTTGCCCTGGCC
	Mm *Icam1* (R)	ACCCTGGGGCAGGAAGGCTT
13	Mm *Ang1* (F)	TGCAAAGGGATGCTCCACACGT
	Mm *Ang1* (R)	AGCATGGTGGCCGTGTGGTTT
14	Mm *Epo* (F)	CCACCCTGCTGCTTTTACTC
	Mm *Epo* (R)	CTCAGTCTGGGACCTTCTGC
15	Mm *Opn* (F)	ACGACCATGAGATTGGCAGT
	Mm *Opn* (R)	GATTCTGCTTCTGAGATGGG
16	Mm *Bmp4* (F)	GCCGCTGAGATCAGGCAGTCC
	Mm *Bmp4* (R)	TGGTCAAAACATTTGCACGTAAAGTC
17	Mm *NCad* (F)	GCCGGAGAGGCACCTGGAGA
	Mm *NCad* (R)	GGTGGTGCCGGTGATGGTGG
18	Mm *Mmp7* (F)	CAGGCCTAGGCGGAGATGCTCA
	Mm *Mmp7* (R)	GCAATGGAGGACCCAGTGAGTGC
19	Mm *Nestin* (F)	GGCAGCAACTGGCACACCTCA
	Mm *Nestin* (R)	GGGAGCACAGATCCCAGGTGC
